# Neutrophil extracellular traps prime the ZBP1-cGAS sensor complex, triggering necroptosis and inflammatory injury in acute pancreatitis

**DOI:** 10.7150/ijbs.122290

**Published:** 2026-02-04

**Authors:** Haoyu Zhang, Zheng Wang, Jiongdi Lu, Jie Li, Yuchen Jia, Xiaozhou Xie, Yixuan Ding, Feng Cao, Fei Li

**Affiliations:** 1 Department of General Surgery, Xuanwu Hospital, Capital Medical University, Beijing, People's Republic of China.; 2 Clinical Center for Acute Pancreatitis, Capital Medical University, Beijing, People's Republic of China.; Haoyu Zhang, M.D., Zheng Wang, M.D. and Jiongdi Lu, M.D. contributed equally to this paper and are co-first authors.

**Keywords:** acute pancreatitis, neutrophil extracellular traps, ZBP1, cGAS, mitochondrial DNA, Necroptosis

## Abstract

Severe acute pancreatitis (SAP) involves dynamic interactions between immune dysregulation and inflammatory infiltration. Although elevated levels of neutrophil extracellular traps (NETs) are associated with SAP, the downstream mechanisms by which NETs exacerbate the inflammatory injury remain unclear. In this study, we demonstrate that NETs levels positively correlate with SAP severity, and pharmacological inhibition of NETs reduces pancreatic injury, and acinar cell death. Mechanistically, NETs activate the ZBP1-cGAS complex via mitochondrial DNA (mtDNA), triggering downstream necroptosis and inflammatory pathways, thereby driving pancreatic inflammatory injury. Specifically, NETs induce mitochondrial damage in acinar cells, leading to cytosolic accumulation of mtDNA. This recruits ZBP1 to form a complex with cGAS dependent on the RHIM domain, wherein ZBP1 stabilizes Z-form mtDNA and potentiates cGAS recognition of Z-mtDNA, thereby cooperatively promoting necroptosis and inflammation. Furthermore, cyclosporine A inhibits mtDNA release, thereby suppressing NETs-induced ZBP1-cGAS complex formation and mitigating pancreatic injury. Our findings establish the mtDNA-ZBP1-cGAS axis as a pivotal mechanism by which NETs exacerbate pancreatic inflammation, revealing new therapeutic targets for SAP.

## 1. Introduction

Severe acute pancreatitis (SAP) represents a life-threatening gastrointestinal emergency characterized by progressive pancreatic necrosis and dysregulated systemic inflammatory response 1, 2. Acinar cell death and the resultant inflammatory cascade serve as defining features of SAP. This pathological cascade drives massive release of pro-inflammatory cytokines that serve as critical mediators of systemic inflammation 3, 4. Acinar cells are not merely passive victims but active participants in this process 5, 6. Acinar cells suffer necrosis by cytokines, releasing additional pro-inflammatory cytokines that exacerbate local injury and perpetuate a self-sustaining inflammatory cycle 7, 8.

Emerging evidence highlights the critical role of immune factors in the molecular pathogenesis of SAP, particularly the study of neutrophil extracellular traps (NETs), which holds significant importance. NETs, released by neutrophils, have been demonstrated to increase in pancreatic infiltration and systemic circulation during AP 9, 10. NETs were reported to directly activate trypsinogen, triggering synchronized necrosis in cells 10-12. Although regulating NETs release has been shown to alleviate SAP progression, the intracellular mechanisms by which NETs damage acinar cells remain poorly understood. It is currently believed that NETs activate cytosolic DNA sensing pathways like cGAS, driving necroptosis in acute lung injury 13. Recent studies revealed that NETs promote ferroptosis and necroptosis by suppressing mitophagy during intestinal epithelial ischemic necrosis 14, 15. This process suggests that NETs may regulate mitochondrial function to activate acinar cell death and inflammatory signaling pathways.

Mitochondrial damage has emerged as a critical research direction in the pathogenesis of sterile inflammation 16, 17. As the cellular powerhouse and largest repository of cytoplasmic DNA, mitochondria actively release mitochondrial DNA (mtDNA) into the cytosol upon injury 18, 19. This released mtDNA triggers inflammatory cascades by activating cytosolic DNA-sensing pathways such as the cGAS pathway 20, 21. Significant advances reveal that oxidative stress induces a conformational shift in mtDNA from B-form to Z-form (Z-mtDNA) 22. Notably, Z-mtDNA exhibits significantly stronger activation potency toward cGAS compared to B-DNA in doxorubicin-induced cardiotoxicity and autoimmune photosensitive diseases and is more readily recognized by Z-DNA binding protein 1 (ZBP1) 22, 23. Consequently, mitochondria function as a signaling hub for cell death and inflammatory responses.

Recent studies have revealed that cytosolic DNA sensors may exhibit synergistic effects in recognizing mtDNA, forming a key amplification mechanism for inflammatory signaling 22, 24. cGAS, the most widely distributed cytosolic DNA sensor, activates downstream inflammatory pathways upon mtDNA recognition 25. Emerging research demonstrates functional crosstalk between ZBP1 and cGAS. ZBP1 recognizes Z-mtDNA via its Zα domain, leading to exposure of its RHIM domain that recruits RIPK3 to drive necroptosis 26, 27. Simultaneously, ZBP1 enhances cGAS recognition of dsDNA through protein-protein interactions, amplifying inflammatory signaling cascades 22, 23. While this cooperative mechanism has been validated in cardiotoxicity models, its role in SAP remains unexplored. Notably, although both cGAS and ZBP1 are individually activated in AP 28, 29, whether their functional interplay exists in SAP represents an uncharted frontier.

This study focuses on the regulation of cytosolic DNA-sensing pathways by NETs. Given that NETs modulate mitochondrial behavior, we hypothesize that: NETs induce mitochondrial damage, leading to mtDNA leakage into the cytosol and subsequent activation of the cGAS pathway. Concurrently, ZBP1 recognizes mtDNA and synergizes with cGAS to drive necroptosis and inflammatory amplification. This study aims to validate that in SAP, NETs mediate acinar cell inflammatory injury by inducing cytosolic mtDNA accumulation and facilitating cGAS-ZBP1 interaction.

## 2. Materials and Methods

### 2.1. Patient recruitment

To investigate the therapeutic significance of NETs in AP, we enrolled patients with AP from the Department of General Surgery, Xuanwu Hospital, Capital Medical University in China. Inclusion criteria comprised adults (aged ≥18 years) with a first episode of AP, defined by the Atlanta Classification 30. Exclusion criteria included: infection, pregnancy, leukemia, chronic pancreatitis, malignancy, prior abdominal surgery and other active acute and chronic inflammation. Twenty patients with mild AP (MAP), 20 with SAP and 20 healthy controls were equally recruited. Blood samples were collected within 24 hours of admission for biomarker analysis. The study protocol was approved by the Ethics Committee of Xuanwu Hospital (2024-183-002), and written informed consent was obtained from all participants. The baseline characteristics of the patients and healthy controls were shown in **Table S1**.

### 2.2. Reagents

Ceruletide (HY-A0190), lipopolysaccharides (LPS, HY-D1056), GSK484 (HY-123606), RU.521 (HY-114180), cGAMP (HY-12512), cyclosporin A (CsA, HY-B0579), Z-VAD (HY-164388), GSK872 (HY-101872), DNase I (HY-108882), Dynasore (HY-15304), phorbol 12-myristate 13-acetate (PMA, HY-18739) were purchased from MCE. Quant-iT™ PicoGreen™ dsDNA assay kit (P7581) was purchased from Thermo Fisher. FastPure cell/tissue DNA isolation kit (DC102-01) was purchased from Vazyme. JC-1 (C2006), mitochondrial permeability transition pore assay kit (C2009), ROS assay kits (DHE: S0064, DCFH-DA: CA1410) and cell counting kit-8 (CCK8, C0037) were purchased from Beyotime. IL6 (EK1222) and TNFα (EK0497) ELISA kits were purchased from SAB. Amylase (C016-1-1) and lipase (A054-1-1) assay kits were purchased from Nanjing Jiancheng. MPO-DNA ELISA kit (ZC-56556) was purchased from ZCiBio. Mitochondrial DNA isolation kit (AB65321) was purchased from Abcam.

The antibodies used in this study were shown in **Table S2**.

### 2.3. Mice and AP models

The study was conducted in accordance with the National Institutes of Health Guide for the Care and Use of Laboratory Animals and approved by the Ethics Committee of Xuanwu Hospital of Capital Medical University (XW20211223-1). C57BL/6 mice (SPF grade, 6 weeks old, 20 ± 2 g) were obtained from WeiTongLiHua Experimental Animal Technical Company (Beijing, China) for AP model establishment. Mice were housed under standard conditions with free access to chow and water, and maintained at 25 °C, 50% relative humidity, and a 12 h light/dark cycle. After 2 weeks of acclimatization, mice underwent a 12 h fasting period prior to experimental procedures.

For model induction, mice received intraperitoneal injections of ceruletide (100 μg/kg) every 1 h for 8 doses. In SAP models, LPS (10 mg/kg) was administered intraperitoneally immediately after the final ceruletide injection, whereas MAP models received equivalent volumes of saline. Control mice were injected with normal saline following the same schedule. At 1 h post-final injection, MAP mice were euthanized, while SAP mice were sacrificed 24 h after the last injection. Pancreatic tissues and serum samples were collected immediately. Pharmacological administration (GSK484, RU.521, cGAMP, CsA, DNase I, Z-VAD, GSK872) was intraperitoneally administered 1 h prior to the first ceruletide injection.

### 2.4. RNA sequencing and bioinformatics analysis

As previously reported, total RNA was isolated from pancreatic tissues with TRIzol reagent. Following quality control assessment, RNA samples underwent online library construction and sequencing. Public transcriptomic dataset GSE194331 was retrieved from the Gene Expression Omnibus (GEO) repository.

Differentially expressed genes (DEGs) were identified through the limma R package using thresholds of P < 0.05 and |log2FC| ≥ 1.0. NETs-related genes (NRGs) were collated from prior studies. Intersection analysis between DEGs and NRGs defined differentially expressed NETs-associated genes (DE-NRGs) in SAP versus Control groups. Functional annotation was performed using clusterProfiler for Gene ontology (GO), Kyoto encyclopedia of genes and genomes (KEGG), and gene set enrichment analysis (GSEA).

### 2.5. Cell model

Under sterile conditions, bone marrow neutrophils were isolated from femurs and tibias of healthy C57BL/6J mice (6-8 weeks old) following euthanasia. Freshly isolated neutrophils were seeded into 6-well plates and cultured in RPMI 1640 medium (Gibco, USA) supplemented with 10% fetal bovine serum (FBS, Umedium, China) at 37 °C in a 5% CO₂ humidified atmosphere for 30 minutes. Subsequently, cells were treated with 100 nM PMA or PBS for 4 hours. After treatment, culture supernatants were discarded, and plates were washed three times with pre-cooled DMEM to elute NETs-containing washes. Cellular debris and intact cells were removed by centrifugation, retaining NETs-rich supernatants. NETs quantities were quantified using PicoGree dsDNA assay kit according to manufacturer protocols.

The mouse pancreatic acinar cell line 266-6 was obtained from ProCell (Danvers, MA) and maintained in DMEM supplemented with 10% FBS. The MAP model was established through 24-hour stimulation with 200 nM ceruletide, while SAP models were developed by co-treating cells with ceruletide and NETs-containing DMEM. Control groups received culture medium containing equivalent solvents. Experimental reagents including Dynasore and CsA were administered 1 hour prior to AP model induction.

### 2.6. Small interfering RNAs (siRNAs), short hairpin RNA (shRNA) and transfection

The sequences of siRNA and shRNA were shown in **Table S3**. SiRNA and plasmid vectors were employed in 266-6 cells. The 266-6 cells were maintained in six-well plates until reaching 70-80% confluency. For transfection, OPTI-MEM reduced-serum medium was added to each well, followed by co-incubation with SiRNA and plasmid vectors and lipofection reagent. Culture medium was replaced with DMEM containing 10% FBS 6 h post-transfection. AP models were established 24 h after transfection, with protein lysates harvested at 48 h for subsequent assays. Other groups were transfected with si-NC or sh-NC as negative administration.

To knock down ZBP1 or introduce a ZBP1 Zα domain mutation in mice, AAV9 carrying sh-Zbp1 (AAV9-sh-Zbp1) or the ZBP1 Zα mutant (Zα^mut^) was used. AAV9 (U6 promoter, 1 × 10¹³ vg/ml, 50 μl) was administered via retrograde pancreatic ductal injection three weeks before establishing the AP model. Control groups received AAV-sh-NC or wild-type (WT) as control. After AP modeling, pancreatic tissues were collected for subsequent experiments.

### 2.7. HE (Hematoxylin and eosin), Immunohistochemistry (IHC) and Immunofluorescence (IF) staining

Pancreatic tissues underwent fixation in 4% paraformaldehyde, paraffin embedding, and serial sectioning into 5-μm-thick slices. HE staining quantified inflammatory infiltration and tissue destruction. Histopathological severity was graded according to Kusske *et al.* 31. For IHC, antigen retrieval was performed on paraffin sections followed by sequential incubation with primary antibodies (overnight, 4 °C) and HRP-conjugated secondary antibodies (1 h, room temperature). Images were captured using an Olympus camera (Tokyo, Japan).

IF was performed on paraffin-embedded tissue sections. Following deparaffinization, rehydration, and antigen retrieval, sections were blocked with 5% sheep serum for 1 hour at room temperature. Subsequently, the sections were incubated with primary antibodies overnight at 4 °C. After washing, they were probed with species-specific Alexa Fluor-conjugated secondary antibodies for 1 hour at room temperature and counterstained with DAPI (1:1000, Abcam) to visualize nuclei. Stained sections were imaged using fluorescence microscopy, confocal microscopy, or lightning super-resolution imaging. DHE staining was used to measure the ROS levels in pancreatic tissue.

### 2.8. Western blot (WB)

Proteins were extracted in RIPA lysis buffer supplemented with PMSF and phosphatase inhibitors. Protein lysates were resolved by SDS-PAGE and transferred to PVDF membranes. Following transfer, membranes were blocked with 5% non-fat milk for 1 hour at room temperature, followed by sequential incubation with primary antibodies (overnight, 4 °C) and HRP-conjugated secondary antibodies (1 hour, room temperature). The bands were visualized using ECL reagents and quantified via densitometry with ImageJ software.

Total protein extracted using cell lysis buffer for co-immunoprecipitation (Co-IP) was incubated with antibodies and magnetic beads (Thermo Fisher, US), and bound proteins were extracted using 5× SDS-PAGE Sample Loading Buffer (Beyotime, China) and detected via WB.

### 2.9. Transmission electron microscopy (TEM)

Pancreatic tissues underwent double fixation in 3% glutaraldehyde and 1% osmium tetroxide, followed by graded acetone dehydration and epoxy resin embedding. Ultrathin sections were prepared via ultramicrotomy, sequentially stained with uranyl acetate and lead citrate, and imaged using a JEM-1400 TEM (JEOL, Japan) at 80 kV.

### 2.10. Biochemical analysis

Circulating free DNA (cfDNA) and MPO-DNA levels were quantified as a marker of NETs release. Serum cfDNA and cytoplasmic dsDNA concentrations were determined using PicoGreen dsDNA kit. MPO-DNA, TNFα and IL6 were quantified using commercially available ELISA kits following manufacturer protocols. Serum amylase and lipase levels were determined via automated biochemical analysis. Pancreatic 266-6 cell viability (CCK-8 assay) were assessed using corresponding colorimetric kits per manufacturer instructions.

### 2.11. Flow cytometry

Flow cytometry protocols included cell death assessment and intracellular ROS quantification. For cell death analysis, cells were processed immediately post-modeling. After washing with pre-chilled PBS, 7-AAD was added and incubated for 15 minutes in the dark prior to flow cytometric detection. Intracellular ROS levels were quantified using a ROS detection kit (DCFH-DA) by flow cytometry following manufacturer instructions.

### 2.12. JC-1 and mPTP staining assays

Mitochondrial membrane potential (MMP) was evaluated using the JC-1 assay kit. Cells were stained with JC-1 dye at 37 °C for 15 minutes under controlled conditions. Post-staining, cells underwent three PBS washes to eliminate residual dye. Fluorescence microscopy was employed to capture emission spectra at 529 nm (green monomeric JC-1) and 590 nm (red J-aggregated JC-1). The methodology for the mPTP staining assays was similar to that described above.

### 2.13 Real-time quantitative polymerase chain reaction (RT-qPCR)

The cytosolic DNA content in 266-6 acinar cells was quantified using qPCR. Cells from each group were equally divided into two aliquots. Cytosolic DNA (with complete removal of nuclear and mitochondrial components) and total DNA were extracted using the FastPure Cell/Tissue DNA Isolation Kit (Vazyme, DC102-01), following a previously described method 32. qPCR amplification was then performed on the cytosolic DNA using specific primers for Cox1, Nd1, D-loop, Mpo and Elane. Simultaneously, nuclear DNA (18s rRNA) was specifically amplified from the total DNA.

The mRNA expression level of Zbp1 was measured using real-time qPCR (RT-qPCR). This process involved extracting mRNA from the cytoplasmic fraction, performing reverse transcription to synthesize complementary DNA (cDNA), followed by quantitative PCR amplification and analysis using specific primers.

The primer sequences are detailed in **Table S2**.

### 2.14. Mitochondrial DNA isolation

Mitochondrial DNA (mtDNA) was extracted from collected 266-6 cells using the Mitochondrial DNA Isolation Kit (AB65321). The process involved isolating mitochondria via differential centrifugation (700×g and 10,000×g) after cell swelling and homogenization, followed by mitochondrial lysis, enzyme treatment, and ethanol precipitation to purify the mtDNA. The resulting mtDNA was used as exogenous mtDNA for subsequent rescue experiments.

### 2.15. Power calculations

Based on prior study 12, the concentration of blood cfDNA is approximately 100 ng/ml in healthy individuals and about 200 ng/ml in AP patients, with a standard deviation of approximately 75. With the parameters set as two-sided alpha = 0.01, a power of 0.9, and a 1:1 ratio between the experimental and control groups, 17 subjects are needed per group. Considering a 15% rate of loss to follow-up and refusal, the planned sample size is approximately 20 subjects per group.

For determining the sample size required per group in experimental acute pancreatitis mice based on histological scoring of pancreatic tissue, preliminary experimental results indicate that the histological score is 2±1 points for control group mice and 11±2 points for the AP group 11, 33. With the parameters set as two-sided alpha = 0.01, a power of 0.9, and a 1:1 ratio between the experimental and control groups, and accounting for a 15% experimental failure rate, the planned sample size is 6 mice per group.

### 2.16. Statistical analysis

Statistical assessments were conducted using GraphPad Prism 8 and R 4.0.3. All experiments in this study were conducted with a minimum of three independent replicates for cellular assays and involved at least six mice for each *in vivo* model. Data are presented as mean ± standard deviation (SD) including WB, IF, flow cytometry. Intergroup comparisons were evaluated using independent samples t-tests. Differences among the three groups were assessed using one-way ANOVA. Spearman's rank correlation analysis quantified associations between the expression of PADI4/CitH3 and pancreatic histopathology scores. The Pearson correlation coefficient (PCC) was utilized to quantify the degree of colocalization in IF analyses, and was performed using the Coloc 2 plugin in Image J software. The band intensities in WB were analyzed and determined using Image J. Statistical significance was defined as *P* < 0.05.

## 3. Results

### 3.1. Inhibition of NETs alleviates acute pancreatic injury in mice

**Figure 1:**
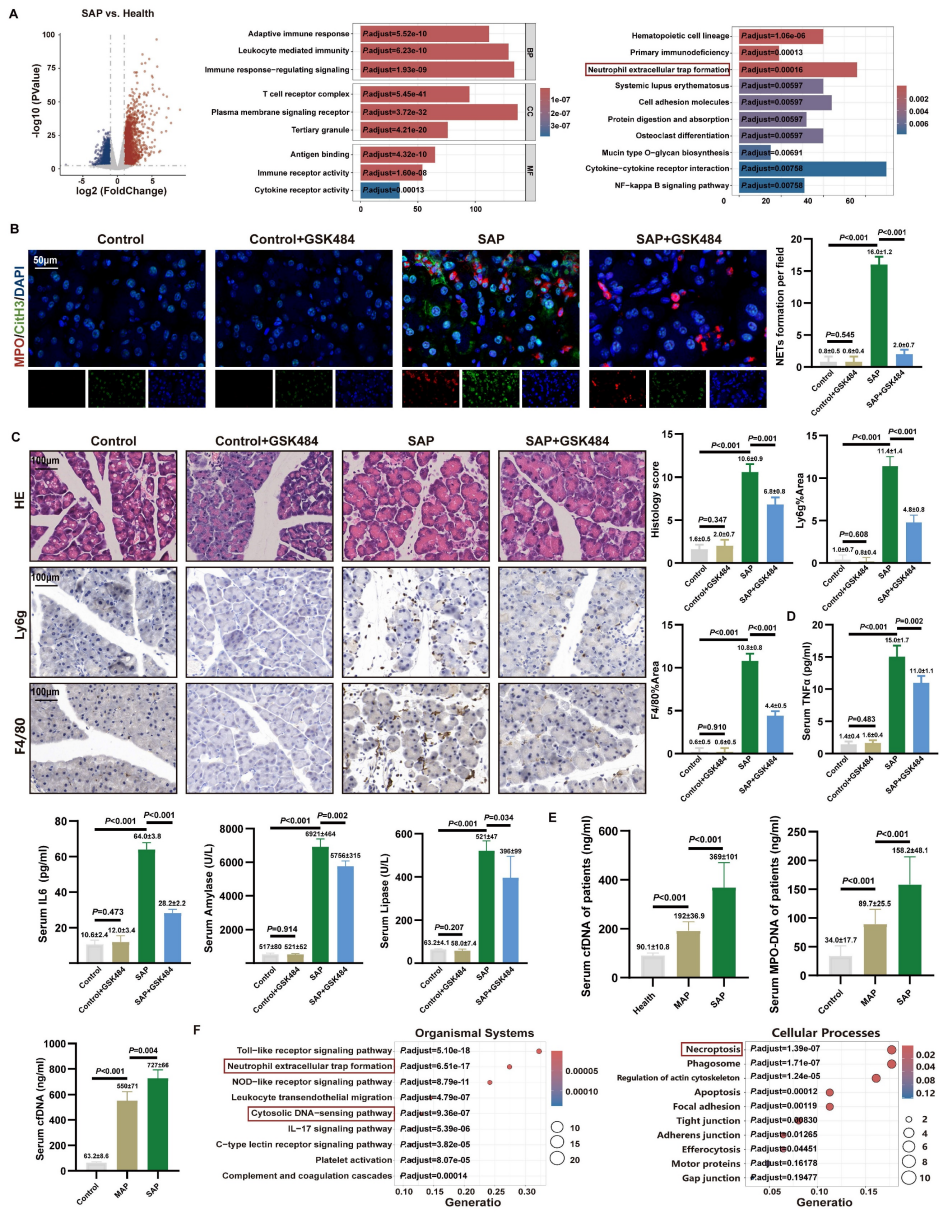
** Inhibition of NETs alleviates acute pancreatic injury in mice.** (A) Differential gene analysis, GO and KEGG enrichment analysis between SAP patients and healthy controls of GSE194331. (B) IF image of CitH3 and MPO of mice pancreas in Control, Control+GSK484, SAP and SAP+GSK484 groups, scale bar=50 μm. (C) HE and IHC staining (Ly6g and F4/80) of mice pancreas in Control, Control+GSK484, SAP and SAP+GSK484 groups, scale bar=100 μm. (D) Expression levels of serum TNFα, IL6, amylase and lipase of mice Control, Control+GSK484, SAP and SAP+GSK484 groups. (E) Serum cfDNA and MPO-DNA level of clinical AP patients and healthy controls, and serum cfDNA level of mice Control, MAP and SAP models. (F) KEGG enrichment analysis of mice pancreatic transcriptome sequencing data. The data are presented as the means ± SD (n=6 mice per group). Intergroup comparisons were evaluated using independent samples t-tests. Statistical significance was defined as* P* < 0.05.

We performed differential gene expression analysis on the RNA sequencing dataset GSE194331 of blood cells from AP patients obtained from the gene expression database (**Figure 1A**). GO analysis revealed that the DEGs were significantly enriched in pathways related to immune cell regulation, while KEGG analysis showed significant enrichment in the “NETs formation” pathway. Analysis comparing SAP patients with MAP patients also demonstrated significant enrichment of DEGs in the “NETs formation” pathway (**Figure S1A**). These findings provided preliminary evidence for the involvement of NETs in AP severity.

To determine the optimal time window for establishing the SAP model, we conducted a comprehensive time-series analysis (**Figure S1B**). Histopathological assessment revealed that pancreatic injury peaked at 24 hours post-injection, characterized by maximal acinar cell damage scores and increased infiltration of neutrophils and macrophages (**Figure S1C**). Consequently, 24 hours after LPS injection was selected as the optimal time point for establishing the SAP model. Furthermore, dynamic monitoring confirmed that the expression levels of key NETs regulatory proteins, PADI4 and CitH3, were positively correlated with AP severity (**Figure S1D**), suggesting a strong association between NETs and disease severity (**Figure S1E**). These findings indicate that NETs formation may be involved in pancreatic injury and the inflammatory cascade during SAP progression.

PADI4 is a histone-modifying enzyme primarily expressed in neutrophils and is crucial for NETs formation 34. We administered the specific PADI4 inhibitor GSK484 (4 mg/kg) to SAP mice, which was previously shown to reduce NETs 11. Visualization of *in situ* NETs formation in pancreatic tissue via CitH3 and MPO staining revealed significant NETs formation in the SAP group, while NETs was markedly reduced upon GSK484 application (**Figure 1B**). WB analysis confirmed that GSK484 treatment significantly inhibited the expression of PADI4 and CitH3 in SAP mice (**Figure S1F**). Histopathological assessment showed that in SAP mice, GSK484 significantly reduced neutrophil/macrophage infiltration and improved acinar cell damaged (**Figure 1C**). Serum levels of TNFα, IL6, amylase, and lipase in SAP mice were also lower in the GSK484 group (**Figure 1D**). We collected serum from clinical SAP and MAP patients and found that serum cfDNA and MPO-DNA concentrations were significantly higher in SAP patients than in MAP patients and healthy controls (**Figure 1E**). Furthermore, serum cfDNA concentration was significantly higher in SAP mice than in MAP mice, and it decreased after GSK484 application (**Figure S1G**). These results indicate that NETs are significant contributor to SAP progression, and inhibiting NETs generation can alleviate SAP severity.

We aimed to elucidate the effect of NETs on pancreatic acinar cells *in vitro*. NETs were generated by stimulating mouse bone marrow-derived neutrophils with PMA, and a cellular AP model was established (**Figure S2A**). Flow cytometry, using a NETs concentration gradient (0-500 ng/ml), showed a dose-dependent positive correlation between cell mortality and NETs concentration, while CCK-8 assays indicated an inverse correlation between cell viability and NETs dose (**Figure S2B**). These findings collectively demonstrate that NETs have a destructive effect on acinar cells *in vitro*.

### 3.2. NETs were involved in cytosolic DNA-sensing pathways in mice

To elucidate the key biological processes driving SAP progression, we performed RNA sequencing on pancreatic tissues from control and SAP model mice. Differential gene analysis identified a total of 4,022 significantly DEGs, including 2,025 upregulated and 1,997 downregulated genes (**Figure S3A**). The top 20 DEGs were predominantly enriched in pathways related to inflammatory infiltration and immune regulation. GSEA further demonstrated significant enrichment of these DEGs in NETs-related pathways (**Figure S3B**).

To clarify the mechanistic link between NETs and pancreatic injury, we integrated 147 NRGs reported in the literature with the SAP-DEGs obtained in this study, identifying 66 overlapping differentially expressed NRGs. KEGG pathway analysis revealed that the cytosolic DNA-sensing pathway was significantly enriched in the organismal systems category, while the regulation of necroptosis was prominently enriched in the cellular processes category (**Figure 1F**). Additionally, GSEA revealed significant enrichment of the type I interferon-mediated signaling pathway and the TNF signaling pathway (**Figure S3B**). These findings suggest that necroptosis and inflammatory infiltration are critical manifestations of NETs-induced pancreatic injury, potentially linked to the accumulation of cytosolic DNA.

NETs are complexes composed of a dsDNA scaffold and proteins. To further validate the disease-promoting role of DNA accumulation in SAP, we administered DNase I, a DNA-specific hydrolase, to SAP group mice. We found that DNase I significantly reduced systemic inflammatory markers TNFα and IL6 in SAP group mice (**Figure S2C**). HE staining showed that DNase I improved acinar destruction with reduced NETs numbers and serum cfDNA level compared to SAP group (**Figure S2D**). This further indicates that the exacerbation of AP pancreatic injury by NETs may be associated with DNA accumulation.

### 3.3. Inhibition of NETs alleviates cGAS pathway activation and necroptosis

**Figure 2:**
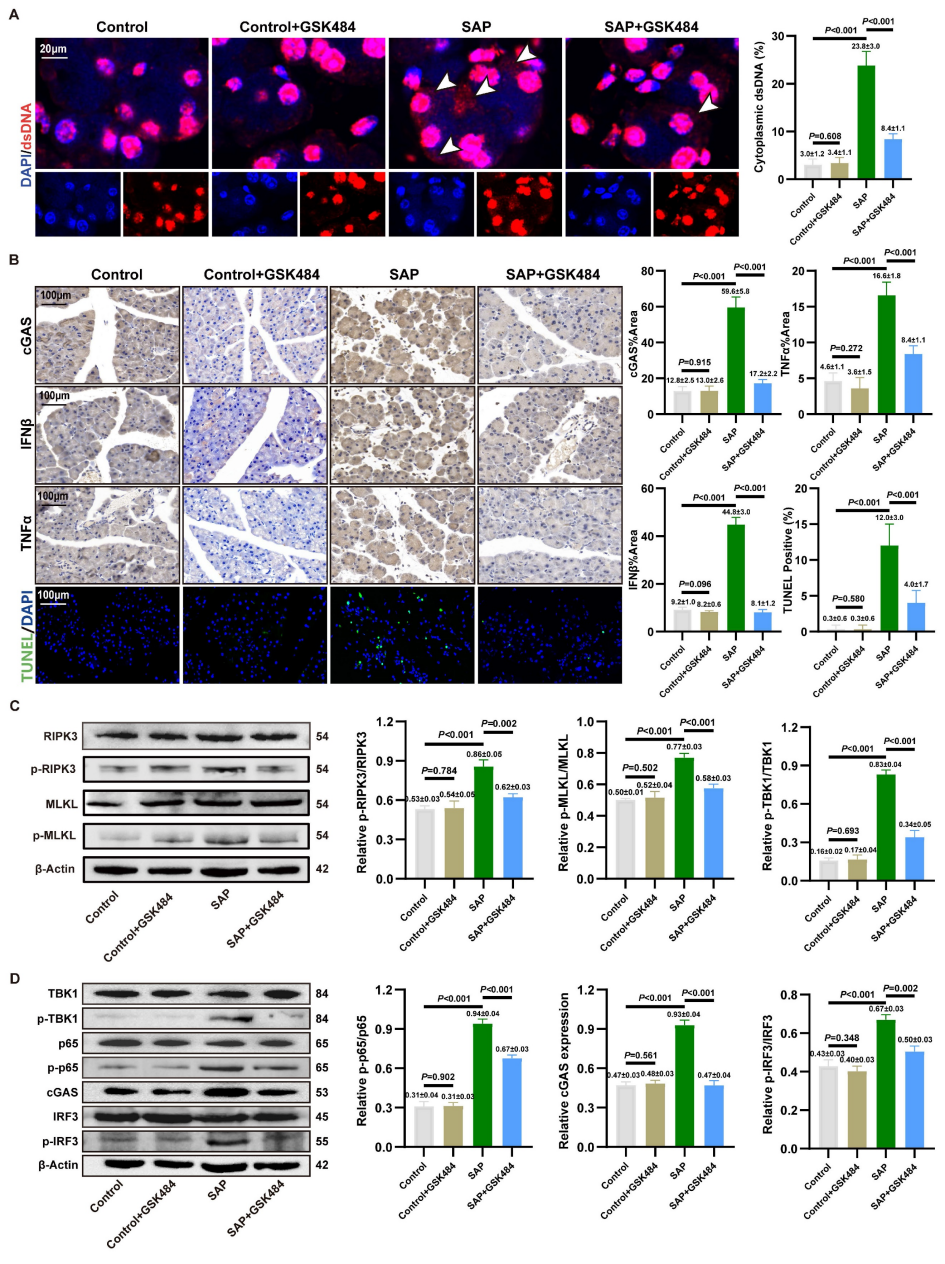
** Inhibition of NETs alleviates cGAS pathway activation and necroptosis.** (A) IF image of dsDNA of pancreas in mice Control, Control+GSK484, SAP and SAP+GSK484 groups, scale bar=20μm. (B) IHC (cGAS, TNFα and IFNβ) and TUNEL staining of pancreas in mice Control, Control+GSK484, SAP and SAP+GSK484 groups, scale bar=100μm. (C) Typical images of WB analyses of p-RIPK3, p-MLKL, RIPK3 and MLKL of pancreas in mice Control, Control+GSK484, SAP and SAP+GSK484 groups. (D) Typical images of WB analyses of cGAS, TBK1, p-TBK1, p65, p-p65, IRF3 and p-IRF3 of pancreas in mice Control, Control+GSK484, SAP and SAP+GSK484 groups. The data are presented as the means ± SD (n=6 mice per group). Intergroup comparisons were evaluated using independent samples t-tests. Statistical significance was defined as* P* < 0.05.

We aimed to clarify the accumulation of cytosolic DNA in acinar cells. IF staining in mouse pancreatic tissues revealed significant localization of dsDNA within the cytoplasm of acinar cells in the SAP group compared to the Control group. Critically, application of GSK484 to inhibit NETs formation markedly reduced the abundance of cytosolic dsDNA (**Figure 2A**). *In vitro*, quantitative analysis of cytoplasmic extracts from 266-6 cells demonstrated that cytosolic dsDNA levels increased significantly by 4 to 5-fold upon NETs stimulation (**Figure S3C**). These results indicate that NETs stimulation likely activates the cytosolic DNA-sensing pathway in acinar cells.

We assessed pancreatic inflammation in mice via IHC staining. IFNβ and TNFα levels were significantly elevated in the acinar cells of SAP mice compared to the Control group, and this increase was reversed by GSK484 treatment (**Figure 2B**). TUNEL staining further indicated that GSK484 significantly reduced the proportion of dead cells in SAP pancreatic tissue. Consistent with RNA-seq results, we examined necroptosis-related proteins. WB analysis confirmed that GSK484 suppressed the expression of p-RIPK3 and p-MLKL in SAP pancreatic tissues (**Figure 2C**).

cGAS is recognized as a primary cytosolic DNA sensor linked to inflammation and cell death. We hypothesized that NETs activate the cGAS pathway. IHC staining confirmed elevated cGAS expression in the acinar cells of SAP mice, which was attenuated by GSK484 (**Figure 2B**). cGAS drives the activation of TBK1, IRF3 and p65, thereby inducing the expression of IFNβ and TNFα. WB analysis showed that GSK484 significantly reduced the levels of cGAS and p-TBK1, p-IRF3, and p-p65 in the pancreas (**Figure 2D**). These findings suggest that NETs promote acinar cell necroptosis and cGAS-mediated pancreatic inflammation in experimental SAP.

We further validated the downstream mechanisms of NETs action on acinar cells *in vitro*. Based on prior findings that NETs possess a dsDNA scaffold and can be internalized via endocytosis 13, we used DNase I and the endocytosis inhibitor Dynasore to block NETs. Furthermore, NETs robustly enhanced cGAS expression and the phosphorylation of TBK1, IRF3, and p65 (**Figure S3D**). Further investigating the downstream effects, NETs were found to amplify the cerulein-induced increase in p-RIPK3 and p-MLKL expression (**Figure S3E**). DNase I treatment reversed the NETs-induced activation of downstream inflammatory and cell death pathways. As shown in **Figure S3F**, both DNase I and Dynasore significantly alleviated the NETs-induced decrease in cell viability. Dynasore also reduced NETs-induced cGAS expression and the activation of TBK1 and MLKL (**Figure S3G**). These results solidify the conclusion that cGAS-driven inflammation and necroptosis, triggered by cytosolic dsDNA accumulation, constitute a key mechanism through which NETs induce acinar cell damage in SAP.

### 3.4. Targeted cGAS pathway influences NETs-mediated pancreatic inflammatory injury

To further elucidate the molecular link between NETs, cGAS pathway activation, and inflammatory injury in pancreatic acinar cells, we used the inhibitor RU.521 and the agonist cGAMP in a mouse SAP model.

**Figure 3:**
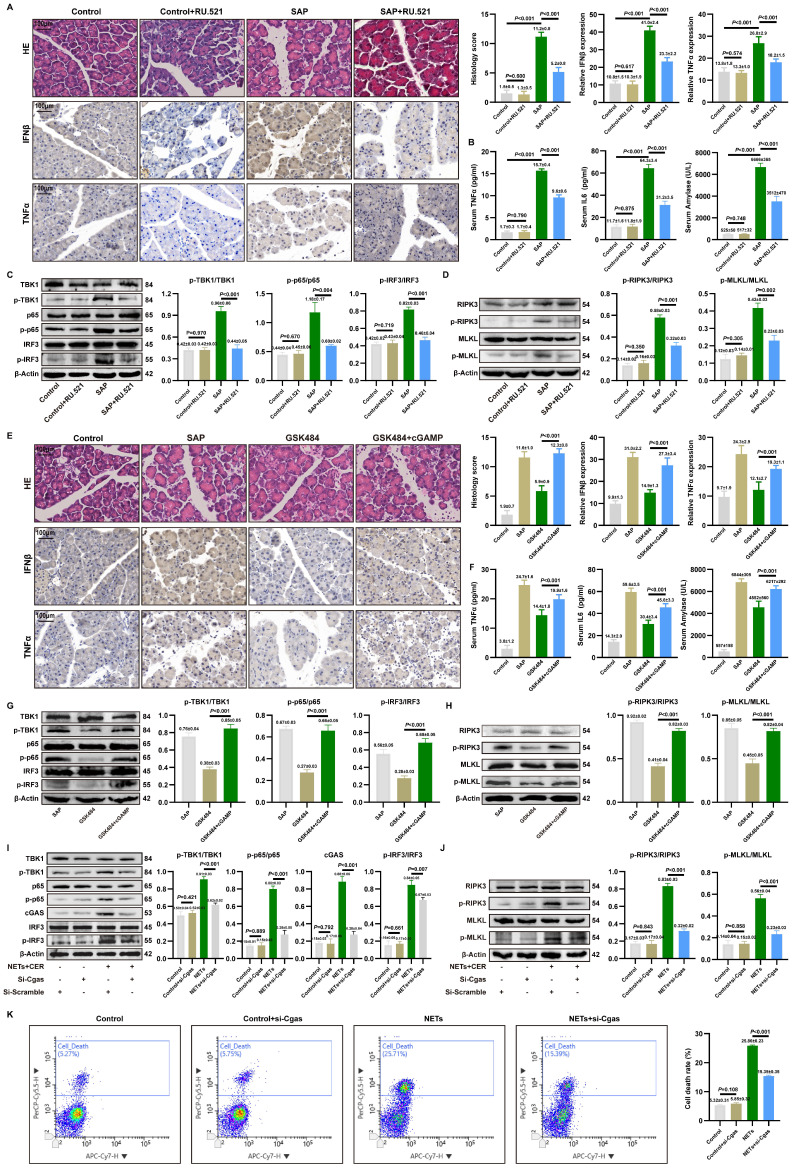
** Targeted cGAS pathway influences NETs-mediated pancreatic inflammatory injury.** (A) HE and IHC staining (TNFα and IFNβ) of pancreas in mice Control, Control+RU52.1, SAP and SAP+RU.521 groups. Scale bar=100μm. (B) Expression levels of serum TNFα, IL6 and amylase in mice Control, Control+RU52.1, SAP and SAP+RU.521 groups. (C) Typical images of WB analyses of TBK1, p-TBK1, p65, p-p65, IRF3 and p-IRF3 of pancreas in mice Control, Control+RU52.1, SAP and SAP+RU.521 groups. (D) Typical images of WB analyses of p-RIPK3, p-MLKL, RIPK3 and MLKL of pancreas in mice Control, Control+RU52.1, SAP and SAP+RU.521 groups. (E) HE and IHC staining (TNFα and IFNβ) of pancreas in mice Control, SAP, GSK484 and GSK484+cGAMP groups. Scale bar=100μm. (F) Expression levels of serum TNFα, IL6 and amylase n mice Control, SAP, GSK484 and GSK484+cGAMP groups. (G) Typical images of WB analyses of TBK1, p-TBK1, p65, p-p65, IRF3 and p-IRF3 of pancreas in mice SAP, GSK484 and GSK484+cGAMP groups. (H) Typical images of WB analyses of p-RIPK3, p-MLKL, RIPK3 and MLKL of pancreas in mice SAP, GSK484 and GSK484+cGAMP groups. (I) Typical images of WB analyses of TBK1, p-TBK1, p65, p-p65, cGAS IRF3 and p-IRF3 in cellular Control, Control+si-Cgas, NETs and NETs+si-Cgas groups. (J) Typical images of WB analyses of p-RIPK3, p-MLKL, RIPK3 and MLKL in cellular Control, Control+si-Cgas, NETs and NETs+si-Cgas groups. (K) The level of cell death in cellular Control, Control+si-Cgas, NETs and NETs+si-Cgas groups. The data are presented as the means ± SD (n=6 mice per group, and n = 3 cells per group). Intergroup comparisons were evaluated using independent samples t-tests. Statistical significance was defined as* P* < 0.05.

RU.521 treatment significantly ameliorated pancreatic histopathological injury, necrosis, immune cell infiltration, reduced acinar cell expression of IFNβ/TNFα (**Figure 3A**). Additionally, serum levels of TNFα, IL6 and amylase were reduced following RU.521 (**Figure 3B**). As shown in **Figure 3C**, RU.521 markedly suppressed the levels of p-TBK1, p-p65, and p-IRF3 in the pancreas of SAP mice. WB analysis further revealed that inhibition of the cGAS downstream pathway reduced the expression of p-RIPK3 and p-MLKL in SAP pancreatic tissues (**Figure 3D**).

Conversely, activating cGAS with cGAMP in mice where NETs were inhibited by GSK484 reversed the protective effects. Assessment of pancreatic injury showed that cGAMP exacerbated pancreatic damage and elevated IFNβ and TNFα expression despite NETs inhibition (**Figure 3E**). Moreover, cGAS pathway activation reversed the reduction in serum TNFα, IL6, and amylase levels (**Figure 3F**). Besides, cGAMP counteracted the GSK484-mediated suppression of p-TBK1, p-p65, and p-IRF3(**Figure 3G**), and increased the expression of p-RIPK3 and p-MLKL (**Figure 3H**), indicating that cGAS is an essential pathway for NETs-regulated necroptosis in acinar cells. These results define the upstream-downstream relationship among NETs, the cGAS pathway, and pancreatic inflammatory injury, revealing that NETs likely induce pancreatic inflammatory injury by modulating the cytosolic DNA-sensing pathway via acinar cell cGAS.

We knocked down cGAS expression in 266-6 cells using si-Cgas and examined its impact on NETs-activated inflammatory pathways and necroptosis. Si-Cgas in 266-6 acinar cells abolished NETs-induced phosphorylation of TBK1, IRF3, and p65 (**Figure 3I**). Furthermore, si-Cgas attenuated the upregulation of necroptotic markers p-RIPK3 and p-MLKL (**Figure 3J**). As shown in** Figure 3K**, si-Cgas transfection did not alter the mortality rate of Control group cells but significantly reduced the high mortality rate caused by NETs. Collectively, these results delineate a signaling axis where NETs act upstream to trigger cGAS-dependent inflammatory and necroptotic signaling, which is the executor of acinar cell damage in SAP.

### 3.5. ZBP1 expression is upregulated by NETs and modulates the cGAS inflammatory pathway

**Figure 4:**
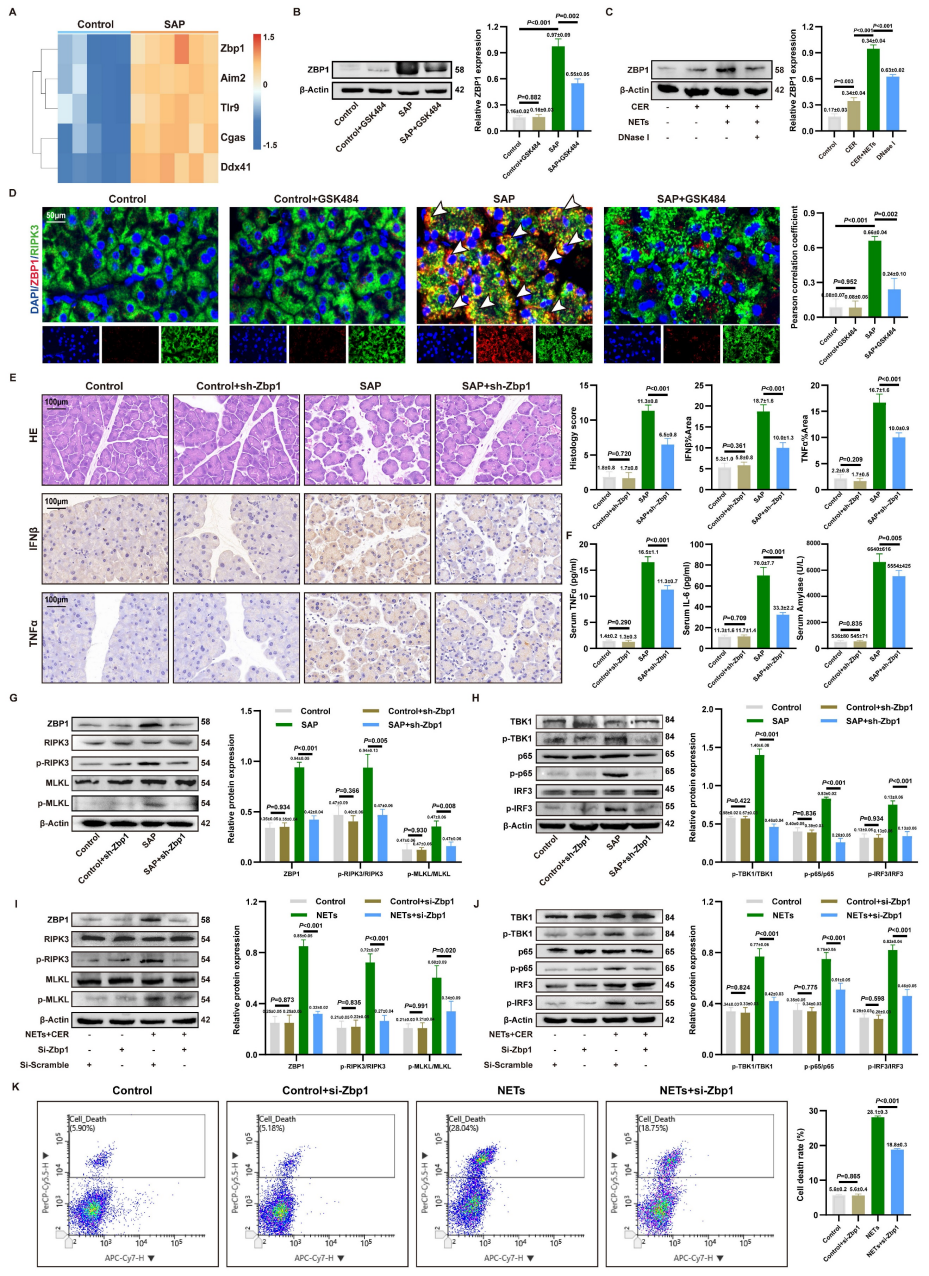
** ZBP1 expression is upregulated by NETs and modulates the cGAS inflammatory pathway.** (A) Heatmap of expression of cytoplasmic DNA sensor in mice pancreatic transcriptome sequencing data. (B) Typical images of WB analyses of ZBP1 of pancreas in mice Control, Control+GSK484, SAP and SAP+GSK484 groups. (C) Typical images of WB analyses of ZBP1 in cellular Control, CER, NETs and DNase I. (D) IF image of ZBP1 and RIPK3 of pancreas in mice Control, Control+GSK484, SAP and SAP+GSK484 groups. Scale bar = 50μm. (E) HE and IHC staining (TNFα and IFNβ) of pancreas in mice Control, Control+sh-Zbp1, SAP and SAP+sh-Zbp1 groups. Scale bar = 50μm. (F) Expression levels of serum TNFα, IL6 and amylase in mice Control, Control+sh-Zbp1, SAP and SAP+sh-Zbp1 groups. (G) Typical images of WB analyses of ZBP1, RIPK3, p-RIPK3, MLKL and p-MLKL in mice Control, Control+sh-Zbp1, SAP and SAP+sh-Zbp1 groups. (H) Typical images of WB analyses of TBK1, p-TBK1, p65, p-p65, IRF3 and p-IRF3 in mice Control, Control+sh-Zbp1, SAP and SAP+sh-Zbp1 groups. (I) Typical images of WB analyses of ZBP1, RIPK3, p-RIPK3, MLKL and p-MLKL in cellular Control, Control+si-Zbp1, NETs and NETs+si-Zbp1 groups. (J) Typical images of WB analyses of TBK1, p-TBK1, p65, p-p65, IRF3 and p-IRF3 in cellular Control, Control+si-Zbp1, NETs and NETs+si-Zbp1 groups. (K) The level of cell death in cellular Control, Control+si-Zbp1, NETs and NETs+si-Zbp1 groups. The data are presented as the means ± SD (n = 6 mice per group, and n = 3 cells per group). Intergroup comparisons were evaluated using independent samples t-tests. Statistical significance was defined as* P* < 0.05.

Since cytosolic DNA sensors often form functional networks through interactions, we evaluated the transcriptional levels of common DNA sensors in SAP. Transcriptomic analysis identified Zbp1 as the most significantly upregulated cytosolic DNA sensor gene in SAP (**Figure 4A**). This upregulation was confirmed at the protein level *in vivo* and shown to be NETs-dependent, as it was reversed by the NETs inhibitor GSK484 (**Figure 4B**). *In vitro*, NETs stimulation induced ZBP1 expression in acinar cells, an effect attenuated by DNase I or the endocytosis inhibitor Dynasore (**Figure 4C**, **Figure S3G**), confirming NETs as ZBP1 inducers.

As ZBP1 is widely documented to directly regulate RIPK3 phosphorylation and necroptosis, ZBP1 co-localized with RIPK3 in SAP acinar cells, and this interaction was disrupted by GSK484 (**Figure 4D**), linking ZBP1 to necroptosis. *In vivo* knockdown of ZBP1 via AAV9-sh-Zbp1 (efficiency confirmed in **Figure S4A-B**) significantly alleviated SAP pathology. This was evidenced by reduced pancreatic injury, lower expression of IFNβ/TNFα in pancreas (**Figure 4E**), decreased serum levels of TNFα, IL6, and amylase (**Figure 4F**), and consistent efficacy across different shRNA sequences (**Figure S4C**). The knockdown did not affect NETs infiltration (**Figure S4D**), positioning ZBP1 downstream of NETs. Mechanistically, ZBP1 knockdown reduced levels of p-RIPK3, p-MLKL (**Figure 4G**) and, notably, also suppressed the activation of the cGAS downstream pathway p-TBK1, p-p65, p-IRF3 in SAP pancreas (**Figure 4H**). This result demonstrates that in NETs-driven cellular injury, ZBP1 not only regulates necroptosis but also participates in modulating cGAS-mediated inflammatory signaling.

We inhibited ZBP1 expression in 266-6 cells via si-Zbp1 transfection. *In vitro*, si-Zbp1 attenuated NETs-promoted p-RIPK3, p-MLKL (**Figure 4I**) and cGAS pathway activation (p-TBK1, p-p65 and p-IRF3) (**Figure 4J**), and reduced NETs-induced cell death (**Figure 4K**). Conversely, ZBP1 overexpression synergized with NETs to further amplify cGAS signaling (**Figure S4E**), an effect blunted by concurrent cGAS knockdown (**Figure S4F**). This demonstrates functional cooperativity between ZBP1 and cGAS downstream of NETs.

Despite the typical antagonism between necroptosis and apoptosis, levels of the apoptotic executioners caspase-8 and cleaved caspase-3 were elevated in SAP (**Figure S5A-B**), suggesting a ZBP1-mediated non-canonical cell death pathway. Functional inhibition experiments revealed that the necroptosis inhibitor GSK872 significantly alleviated pancreatic injury, whereas the apoptosis inhibitor Z-VAD had no significant effect (**Figure S5C**), underscoring the critical pathogenic role of the ZBP1-necroptosis axis in SAP.

### 3.6. NETs activate the formation of a ZBP1-cGAS sensor complex

**Figure 5:**
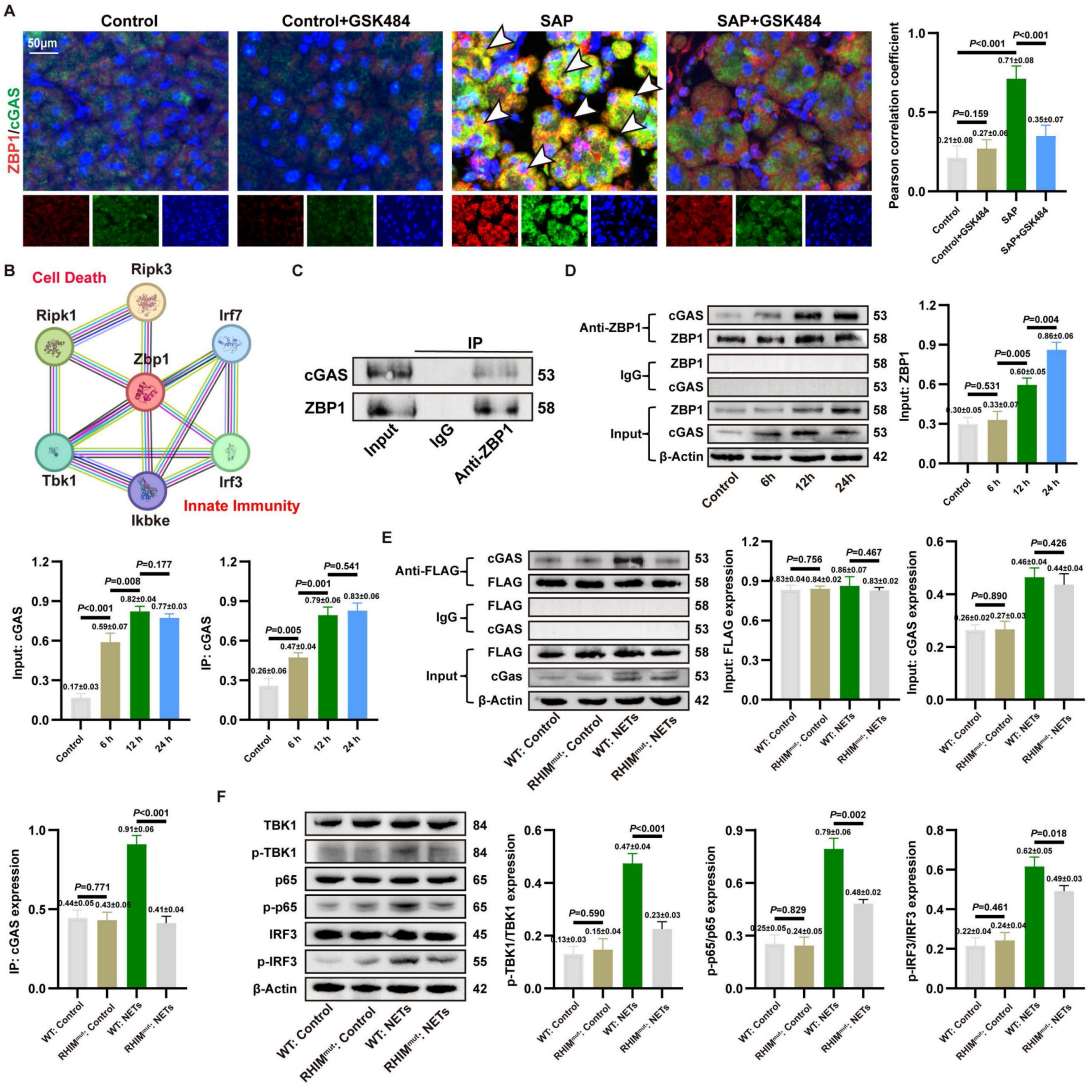
** NETs activate the formation of a ZBP1-cGAS sensor complex.** (A) IF image of ZBP1 and RIPK3 of pancreas in mice Control, Control+GSK484, SAP and SAP+GSK484 groups. Scale bar = 50μm. (B) Protein-molecule interaction network of Zbp1. (C) Co-ip of ZBP1 and cGAS of 266-6 cells in AP models. (D) Co-ip of ZBP1 and cGAS in 266-6 cells at 6, 12, and 24 hours after the induction of AP model. (E) Co-ip of ZBP1 and cGAS of 266-6 cells after the mutation of RHIM domain. (F) Typical images of WB analyses of TBK1, p-TBK1, p65, p-p65, IRF3 and p-IRF3 of 266-6 cells after the mutation of RHIM domain. The data are presented as the means±SD (n = 6 mice per group, and n = 3 cells per group). Intergroup comparisons were evaluated using independent samples t-tests. Statistical significance was defined as* P* < 0.05.

To investigate the interaction between ZBP1 and cGAS in SAP, we performed IF co-staining. The results showed significantly enhanced co-localization of ZBP1 and cGAS in pancreatic acinar cells of SAP mice compared to controls, which was markedly attenuated by GSK484-mediated NETs inhibition (**Figure 5A**). STRING database analysis also suggested close associations between ZBP1 and cGAS downstream signaling molecules (**Figure 5B**). Combined with co-IP assays in NETs-stimulated 266-6 cells confirming a direct and strong ZBP1-cGAS interaction (**Figure 5C**), these findings indicate that ZBP1 and cGAS likely form a functional complex in NETs-induced SAP, beyond mere synergy.

We analyzed the signaling chronology during SAP progression. As shown in **Figure S6A**, IHC revealed that pancreatic levels of TNFα and IFNβ began to rise significantly at 6 hours, peaked at 12-24 hours, and declined by 48 hours, which was aligns with the established progression of tissue damage. Further WB revealed that cGAS and its downstream signals p-p65 and p-IRF3 were activated early (2h), while upregulation of ZBP1 and p-MLKL was delayed (6h), suggesting cGAS activation precedes ZBP1 upregulation (**Figure S6B**). *In vitro* assays confirmed that cGAS activation (6h) occurred earlier than ZBP1 upregulation (12h) post-NETs stimulation, yet a strong interaction persisted throughout (**Figure 5D**). Notably, ZBP1 knockdown significantly suppressed the sustained increase in NETs-induced downstream signals p-TBK1, p-MLKL (**Figure S6C**), indicating a positive feedback loop: early cGAS activation induces ZBP1, which in turn amplifies cGAS signaling.

Prior studies suggested ZBP1 might bind cGAS via its RHIM domain 22. We validated this using a ZBP1 RHIM domain mutant. The mutant showed significantly reduced binding to cGAS (**Figure 5E**). Functionally, the RHIM mutation also impaired phosphorylation of downstream p-TBK1, p-p65, and p-IRF3 (**Figure 5F**). Collectively, these results demonstrate that NETs promote the formation of a RHIM domain-dependent ZBP1-cGAS complex in acinar cells. This complex serves as a positive feedback core that drives inflammatory signal amplification, exacerbating pancreatic injury.

### 3.7. NETs induce mitochondrial damage and cytosolic mtDNA accumulation in acinar cells

**Figure 6:**
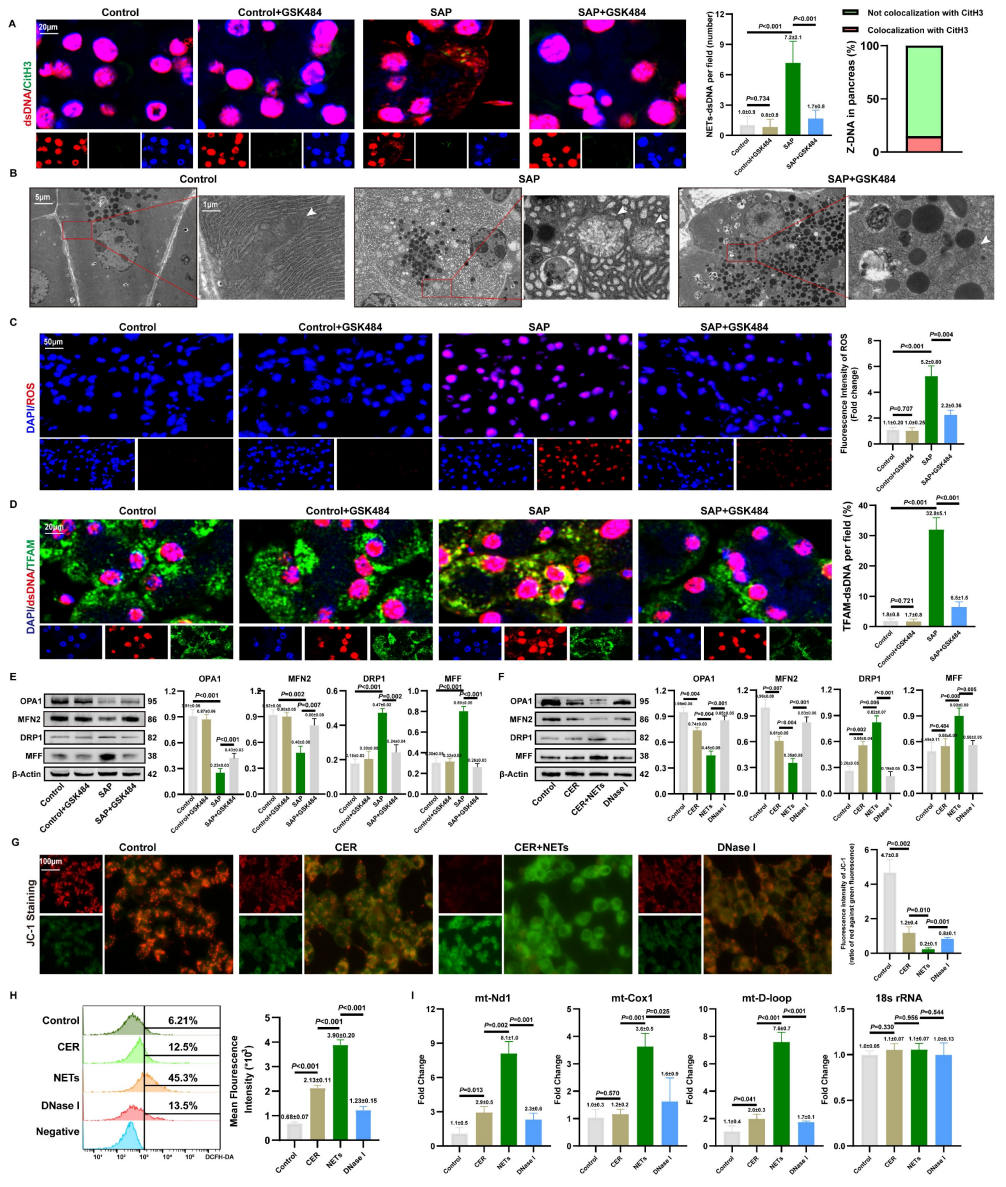
** NETs induce mitochondrial damage and cytosolic mtDNA accumulation in acinar cells.** (A) IF image of CitH3 and dsDNA of pancreas in mice Control, Control+GSK484, SAP and SAP+GSK484 groups. Scale bar = 20μm. (B) Transmission electron microscopy images of mitochondrion of pancreas in mice Control, SAP and SAP+GSK484 groups. White arrow: mitochondria. Scale bar = 5μm and 1μm. (C) ROS levels of mice pancreas by DHE. Scale bar = 50μm. (D) IF image of dsDNA and TFAM of pancreas in mice Control, Control+GSK484, SAP and SAP+GSK484 groups. Scale bar = 20μm. (E) Typical images of WB analyses of OPA1, MFN2, DRP1 and MFF of pancreas in mice Control, Control+GSK484, SAP and SAP+GSK484 groups. (F) Typical images of WB analyses of OPA1, MFN2, DRP1 and MFF in cellular Control, CER, NETs and DNase I gourps. (G) Mitochondrial membrane potential levels in 266-6 cells by JC-1 staining in cellular Control, CER, NETs and DNase I gourps. Scale bar = 50μm. (H) ROS levels of 266-6 cells by flow cytometry in cellular Control, CER, NETs and DNase I gourps. (I) The copy numbers of cytosolic mt-Nd1, mt-Cox1, and mt- D-loop of cellular Control, CER, NETs and DNase I gourps. The data are presented as the means±SD (n = 6 mice per group, and n = 3 cells per group). Intergroup comparisons were evaluated using independent samples t-tests. Statistical significance was defined as* P* < 0.05.

To investigate the source of cytosolic dsDNA induced by NETs, we first assessed the condition of the NETs' DNA backbone. As shown in **Figures 6A** and **S7A**, we observed co-localization of cytosolic dsDNA with CitH3 in acinar cells. qPCR analysis showed that NETs stimulation increased the copy numbers of Mpo and Elane in the cytoplasm, which decreased after DNase I treatment (**Figure S7B**). Combined with the inability of DNase I-treated NETs to induce cytosolic dsDNA accumulation, these findings suggest that acinar cells can internalize NETs along with their DNA scaffold.

However, as shown in **Figure 6A**, the limited local co-localization of CitH3 with dsDNA indicated that NETs is not the primary source activating the DNA-sensing pathway. Based on clues that NETs can regulate mitochondrial function, we further explored their impact on mitochondria. TEM revealed severe mitochondrial swelling, cristae fragmentation, and other damage in the pancreas, which was partially ameliorated by NETs inhibition (**Figure 6B**). DHE staining confirmed elevated ROS levels in SAP tissues, an effect reversible by NETs inhibition (**Figure 6C**). IF showed significantly increased co-localization of cytosolic dsDNA with the mitochondrial protein TFAM in SAP acinar cells, which was attenuated by NETs inhibition (**Figure 6D**), strongly suggesting a mitochondrial origin of the dsDNA. WB further demonstrated that GSK-mediated NETs inhibition reduced mitochondrial fission proteins DRP1 and MFF, while increasing fusion proteins OPA1 and MFN2 (**Figure 6E**).

*In vitro* studies validated these findings. WB indicated that NETs upregulate DRP1 and MFF, and downregulate OPA1 and MFN2 (**Figure 6F**). JC-1 staining showed that NETs stimulation significantly reduced red fluorescence intensity and increased green fluorescence, indicating mitochondrial membrane potential collapse (**Figure 6G**). Flow cytometry revealed increased ROS levels in NETs-stimulated cells (**Figure 6H**), confirming that NETs promote excessive ROS production. qPCR analysis showed increased copy numbers of mitochondrial genes mt-Nd1, mt-Cox1, and mt-D-loop in NETs-stimulated 266-6 cells compared to controls (**Figure 6I**). Together, these results indicate that NETs induce mitochondrial damage and mtDNA leakage in acinar cells, thereby activating the cytosolic DNA-sensing pathway.

We also examined the spatial relationship between NETs and mitochondria in acinar cells both *in vivo* and *in vitro*. As shown in **Figures S7C-D**, CitH3 co-localized with TOMM20. Furthermore, NETs stimulation significantly increased mPTP fluorescence intensity in acinar cells, which decreased after DNase I treatment (**Figure S7E**). This suggests that NETs potentially cause mitochondrial damage, with mtDNA released into the cytoplasm via mPTP. Given that NETs are rich in positively charged histones, we used strongly negatively charged heparin for neutralization. JC-1 staining showed that heparin significantly alleviated NETs-induced MMP collapse (**Figure S7F**), and ROS levels were markedly reduced (**Figure S7G**). These results further strengthen the evidence that NETs can damage mitochondria in acinar cells.

### 3.8. ZBP1-cGAS complex binds to mtDNA

**Figure 7:**
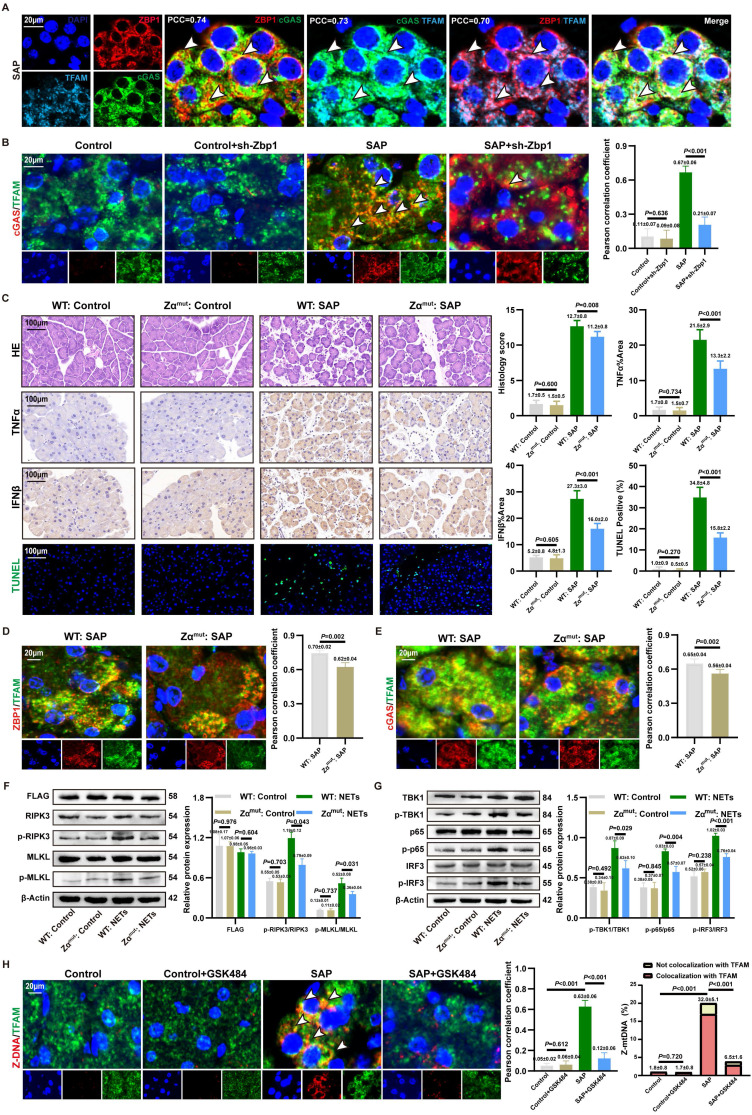
** ZBP1-cGAS complex binds to mtDNA.** (A) IF image of ZBP1, cGAS and TFAM of mice pancreas of SAP group. Scale bar = 20μm. (B) IF image of cGAS and TFAM of pancreas in mice Control, Control+sh-Zbp1, SAP, SAP+sh-Zbp1 groups. Scale bar = 20μm. (C) HE, IHC (TNFα and IFNβ) and TUNEL staining of pancreas in mice WT: Control, Zα^mut^: Control, WT: SAP and Zα^mut^: SAP groups. Scale bar = 100μm. (D) IF image of ZBP1 and TFAM of pancreas in mice WT: SAP and Zα^mut^: SAP groups. Scale bar = 20μm. (E) IF image of cGAS and TFAM of pancreas in mice WT: SAP and Zα^mut^: SAP groups. Scale bar =20μm. (F) Typical images of WB analyses of FLAG, p-RIPK3, p-MLKL, RIPK3 and MLKL in cellular WT: Control, Zα^mut^: Control, WT: NETs and Zα^mut^: NETs groups. (G) Typical images of WB analyses of TBK1, p-TBK1, p65, p-p65, IRF3 and p-IRF3 in cellular WT: Control, Zα^mut^: Control, WT: NETs and Zα^mut^: NETs groups. (H) IF image of Z-DNA and TFAM of pancreas in mice Control, Control+GSK484, SAP, SAP+GSK484 groups. Scale bar = 20μm. The data are presented as the means±SD (n=6 mice per group, and n=3 cells per group). Intergroup comparisons were evaluated using independent samples t-tests. Statistical significance was defined as* P* < 0.05.

MtDNA is a key activator of ZBP1 and cGAS 17, 35. IF revealed significant cytoplasmic co-localization of ZBP1, cGAS, and TFAM (**Figure 7A and S8A**), indicating that the NETs-induced ZBP1-cGAS complex likely associates with mtDNA. To investigate whether ZBP1 affected the binding of cGAS to mtDNA, we observed a significantly reduced co-localization of cGAS with TFAM in SAP+sh-Zbp1 mice (**Figure 7B**), demonstrating that ZBP1 promotes the anchoring of cGAS to mtDNA.

The Zα domain is critical for ZBP1 to bind mtDNA and become activated 36. To elucidate the mechanism by which ZBP1 facilitates cGAS-mtDNA interaction, we constructed a ZBP1 Zα domain mutant *in vivo*. Under conditions of comparable viral transduction efficiency, Zα^mut^:SAP mice exhibited significantly alleviated pancreatic acinar structure damage, lower expression of TNFα and IFNβ in tissues, and a reduced proportion of cell death by TUNEL staining compared to WT: SAP mice, with no significant difference in NETs infiltration between the groups (**Figure 7C and S8B**). This underscores the essential role of ZBP1's Zα domain in NETs-induced pancreatic injury. Mechanistically, the Zα domain mutation not only significantly reduced the co-localization of ZBP1 itself with TFAM but also impaired the association between cGAS and TFAM (**Figure 7D-E**). *In vitro* experiments further confirmed that in cells with the Zα domain mutation, NETs stimulation-induced phosphorylation levels of necroptosis markers (p-RIPK3, p-MLKL) (**Figure 7F**) and cGAS downstream inflammatory signaling molecules (p-TBK1, p-p65, p-IRF3) (**Figure 7G**) were substantially lower than those in WT: SAP group. These results indicate that ZBP1 binding to mtDNA via its Zα domain is a prerequisite for the effective activation of the cGAS pathway.

We further explored the mechanism by which ZBP1 promotes cGAS binding to mtDNA. ZBP1 is known to be activated by Z-DNA 37. IF revealed abundant Z-DNA signals co-localizing with TFAM in the cytoplasm of damaged acinar cells (**Figure 7H**), identified as Z-form mtDNA (Z-mtDNA), which was reduced in GSK484-treated groups and validated in *in vitro* NETs stimulation models (**Figure S8A**), indicating that mitochondrial stress induced by NETs promotes Z-mtDNA accumulation. Consistent with prior study reporting ZBP1's role in stabilizing cytosolic Z-mtDNA 22, we found that the co-localization signal between TFAM and Z-DNA was significantly weakened both in *in vivo* and *in vitro* models with ZBP1 Zα domain mutation (**Figure S8C**), as well as in sh-Zbp1 SAP mice (**Figure S8D**). This demonstrates that ZBP1 specifically recognizes and stabilizes NETs-induced Z-mtDNA. Notably, under SAP conditions, cGAS also showed extensive co-localization with cytosolic Z-DNA (**Figure S8E**). We propose that ZBP1, by binding to Z-mtDNA, recruits cGAS to form a functional complex, thereby driving a positive feedback loop that amplifies downstream inflammatory and necroptotic signaling.

### 3.9. CsA reverses NETs-mediated ZBP1-cGAS complex formation and pancreatic injury

CsA could inhibit mtDNA release into the cytoplasm 18. To validate the role of cytosolic mtDNA in the activation of the NETs-induced inflammatory injury, we applied CsA in SAP models.

**Figure 8:**
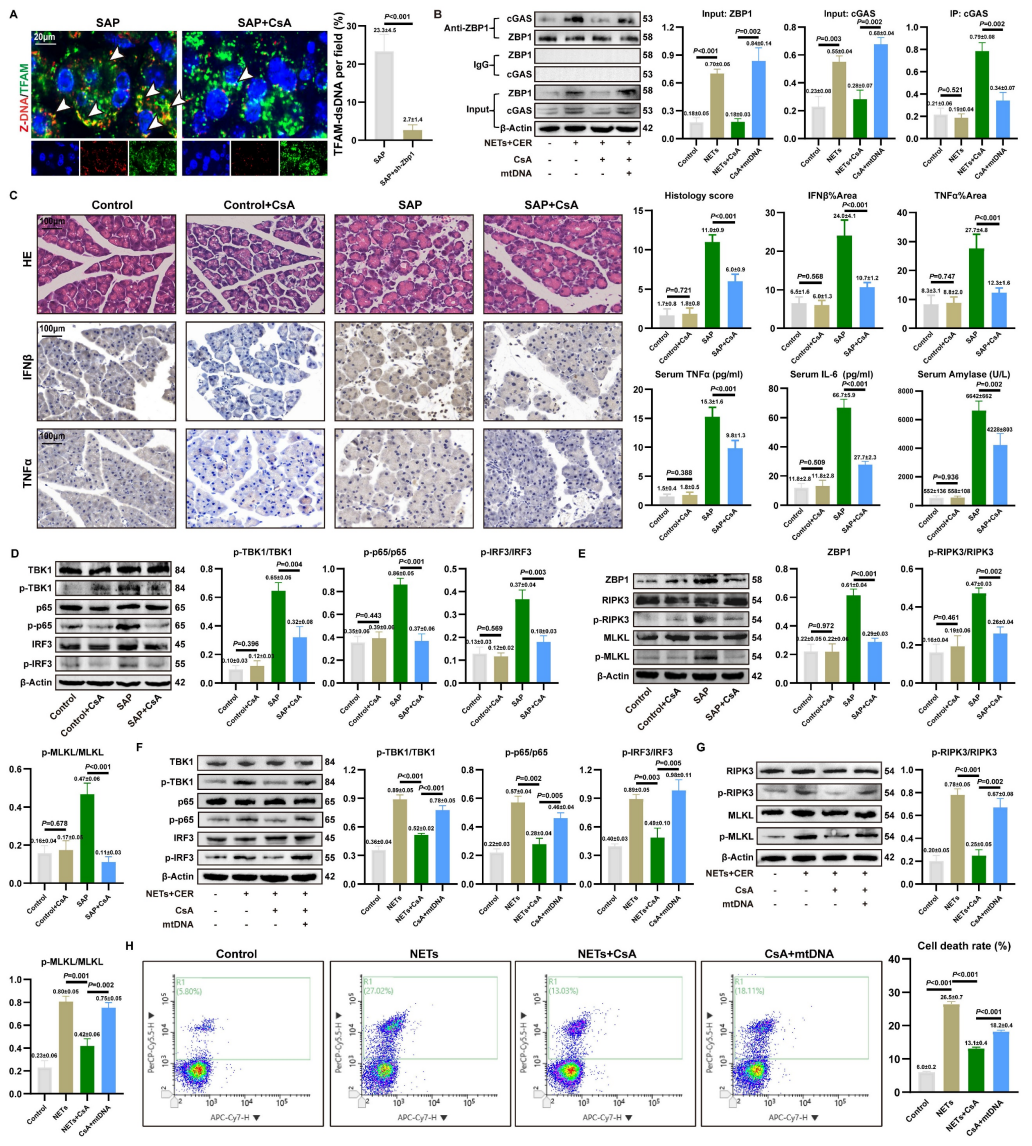
** CsA reverses NETs-mediated ZBP1-cGAS complex formation and pancreatic injury.** (A) IF image of Z-DNA and TFAM of pancreas in mice SAP and SAP+CsA. Scale bar=20μm. (B) Co-ip of cGAS and ZBP1 in cellular Control, NETs, NETs+CsA and CsA+mtDNA groups. (C) HE and IHC staining (TNFα and IFNβ) of pancreas, and expression levels of serum TNFα, IL6 and amylase in mice Control, Control+CsA, SAP, SAP+CsA groups. Scale bar=100μm. (D) Typical images of WB analyses of TBK1, p-TBK1, p65, p-p65, IRF3 and p-IRF3 of pancreas in mice Control, Control+CsA, SAP, SAP+CsA groups. (E) Typical images of WB analyses of ZBP1, RIPK3, p-RIPK3, MLKL and p-MLKL of pancreas in mice Control, Control+CsA, SAP, SAP+CsA groups. (F) Typical images of WB analyses of TBK1, p-TBK1, p65, p-p65, IRF3 and p-IRF3 in cellular Control, NETs, NETs+CsA and CsA+mtDNA groups. (G) Typical images of WB analyses of RIPK3, p-RIPK3, MLKL and p-MLKL in cellular Control, NETs, NETs+CsA and CsA+mtDNA groups. (H) The level of cell death in cellular Control, NETs, NETs+CsA and CsA+mtDNA groups. The data are presented as the means±SD (n=6 mice per group, and n=3 cells per group). Intergroup comparisons were evaluated using independent samples t-tests. Statistical significance was defined as* P* < 0.05.

As shown in **Figure 8A**, CsA treatment significantly reduced the accumulation of Z-mtDNA in the acinar cells of SAP mice. Co-IP further demonstrated that CsA attenuated the NETs-induced interaction between ZBP1 and cGAS, and this disrupted interaction was restored by exogenous mtDNA supplementation (**Figure 8B**). This confirms that the assembly of the NETs-driven ZBP1-cGAS complex depends on mtDNA release.

We further evaluated the protective effect of CsA against pancreatic injury in SAP. CsA treatment did not significantly alter the extent of NETs infiltration in pancreatic tissue (**Figure S9A**) or the expression of key NETs formation proteins PADI4 and CitH3 (**Figure S9B**). Importantly, CsA administration markedly alleviated pancreatic histopathological damage and infiltration of pro-inflammatory cytokines in SAP mice (**Figure 8C**), and also reduced serum levels of TNFα, IL6, and amylase.WB revealed that CsA treatment significantly decreased the level of p-TBK1, p-p65, and p-IRF3 (**Figure 8D**), and concurrently reduced the expression of necroptosis markers p-RIPK3 and p-MLKL (**Figure 8E**). These findings are consistent with the observed decrease in pro-inflammatory cytokine levels in the tissues.

*In vitro* experiments using NETs-stimulated 266-6 acinar cells corroborated these findings. CsA inhibited the NETs-induced phosphorylation of TBK1, p65, and IRF3 (**Figure 8F**) and attenuated NETs-promoted necroptosis (**Figure 8G**). Notably, the inhibitory effects of CsA on these inflammatory and cell death signaling pathways were reversed by the addition of exogenous mtDNA. Furthermore, CsA significantly reduced the mortality of NETs-stimulated acinar cells, and this protective effect was similarly counteracted by exogenous mtDNA (**Figure 8H**). These results demonstrate that cytosolic mtDNA is involved in NETs-mediated pro-inflammatory cytokine release and acinar cell necroptosis. CsA alleviates NETs-driven pancreatic inflammatory injury by inhibiting mtDNA release.

## 4. Discussion

Immune-mediated pancreatic inflammatory injury in AP has garnered increasing attention. Due to the complex molecular mechanisms underlying SAP, limited pharmacological options are available to alleviate pancreatic inflammation. In this study, we demonstrated elevated circulating NETs in SAP patients. Experimental AP models revealed that NETs mediate pancreatic inflammatory injury through cytosolic DNA-sensing pathway regulation. These findings support the detrimental role of NETs in pancreatic inflammation during SAP. Our study uncovered a novel mechanism: NETs induced mitochondrial damage, and activated the mtDNA-ZBP1-cGAS complex interaction thereby exacerbating acinar cell inflammatory injury.

NETs, as extracellular structures released by neutrophils, exhibit gene enrichment patterns that not only serve as molecular evidence of neutrophil activation but also act as systemic “signal amplifiers” for pancreatic NETs infiltration 9. Merza *et al.* demonstrated that histones H3 and H4 within NETs directly activate trypsinogens, inducing acinar cell cytotoxicity 12. AP is fundamentally a bidirectional inflammatory disease driven by pancreatic injury and immune infiltration, wherein hyperactivation of trypsin plays a central pathogenic role 38. NETs exacerbate this pathological process by further amplifying trypsin activity, thereby serving as a pivotal mechanism for disease progression.

NETs activate the cytosolic DNA-sensing pathway via cGAS, thereby promoting necroptosis and inflammation in acinar cells. Pathologically, AP is characterized by uncontrolled inflammatory responses triggered by acinar cell injury, where cross-activation of multiple inflammatory pathways forms an amplification network that reciprocally drives acinar cell death 6, 8. In SAP models, NETs enhance cGAS expression while concurrently activating downstream pro-inflammatory and necroptosis—a finding corroborated by both *in vivo* and *in vitro* experiments. cGAS occupies a central role in regulating inflammation and cell death in SAP, as targeting cGAS function and expression significantly alters NETs-induced necroptosis and pro-inflammatory cytokine expression.

NETs were demonstrated to enhance ZBP1 expression during SAP pathogenesis. ZBP1 phosphorylates RIPK3, establishing its role as a key node in NETs-mediated necroptosis. Notably, as a classical interferon-stimulated gene, ZBP1 also reciprocally regulates cGAS signaling activation and pro-inflammatory cytokine release. While ZBP1-cGAS complex formation was previously reported in doxorubicin-induced cardiotoxicity 22, we confirm its existence in SAP and demonstrate that this interaction—independent of downstream cGAS signaling—depends on the RHIM domain of ZBP1. The ZBP1-cGAS complex suggests a shared regulatory mechanism bridging necroptotic and inflammatory pathways. We propose that this complex is a distinct molecular signature generated intracellularly in acinar cells upon NETs stimulation, as both DNA sensors are transcriptionally upregulated by NETs.

Notably, the primary source of cytoplasmic dsDNA in acinar cells is mtDNA rather than DNA backbone of NETs. The regulatory role of NETs in mitochondrial dynamics and oxidative stress has been well-established. For instance, in intestinal epithelial injury, NETs promote ferroptosis by suppressing mitophagy 14. Similarly, NETs are implicated in ROS-mediated pathologies in Kawasaki disease and sepsis 39, 40. Mechanistically, both ZBP1 and cGAS, as DNA sensors, were found to bind mtDNA. This suggests that NETs create a cytoplasmic dsDNA-enriched microenvironment through mitochondrial damage, providing a platform for ZBP1-cGAS complex formation and activation. Our functional experiments suggest that NETs may directly target acinar cell mitochondria, although the precise mechanism remains elusive. Existing study indicates that histones within NETs can disrupt membrane integrity via their positive charge or activate trypsin 12. Therefore, determining whether the primary mechanism involves the direct action of histones or proteases will be crucial for elucidating the core events of organelle damage in pancreatitis. This constitutes an important direction for future research.

We demonstrate that the functionality of the NETs-activated ZBP1-cGAS cytosolic DNA sensor complex depends on mtDNA. We confirmed co-localization and physical interactions among cGAS, ZBP1, and the mtDNA marker TFAM. Notably, cGAS binds to ZBP1 and is similarly activated by Z-mtDNA. MtDNA undergoes a transition to form Z-form under oxidative stress, which we verified accumulates extensively in the cytoplasm of acinar cells both *in vivo* and *in vitro*. As reported in autoimmune photosensitivity contexts, Z-DNA exhibits stronger cGAS-activating capability than canonical B-DNA 23. Mechanistically, analogous to observations in doxorubicin-induced cardiotoxicity 22, ZBP1 stabilizes the Z-conformation of mtDNA and recruits cGAS to form a functional complex. Similarly, in SAP, ZBP1, cGAS, and mtDNA constitute a tripartite interaction network. Genetic knockdown of ZBP1 or mutation of its Zα domain impairs cGAS recognition of Z-form mtDNA. These findings collectively reveal a pathogenic axis termed the “NETs-mtDNA-ZBP1-cGAS axis”.

The limitations of our study should be mentioned. While our study provides initial evidence for NET-mediated acinar cell injury mechanisms, the therapeutic efficacy of targeting downstream inflammatory pathways in clinical SAP remains speculative. The translational gap between preclinical findings and human pathophysiology necessitates rigorous validation through additional basic research and clinical trials. These future investigations will be critical to refining NET-targeted therapies and establishing their safety profiles in SAP management. Besides, despite multiple lines of evidence supporting the mitochondrial origin of Z-DNA, including its co-localization with TFAM and functional validation using ZBP1 Zα domain mutants, this study lacks direct sequencing-based proof of Z-form conformation within mtDNA. The development of mtDNA-specific Z-form sequencing technologies in the future will be crucial to provide definitive evidence for the existence of Z-mtDNA.

## 5. Conclusion

In summary, our study demonstrates that in SAP, NETs induce mitochondrial damage, leading to the accumulation of mtDNA in the cytoplasm, which regulates the formation of the ZBP1-cGAS complex. By promoting cGAS recognition of mtDNA, ZBP1 enhances the activation of cGAS-dependent inflammatory pathways, establishing a pathological positive feedback loop involving sterile inflammation and necroptosis. These findings underscore that NETs are not only a key initiating factor in SAP exacerbation but also provide a novel mechanistic framework for developing targeted therapeutic strategies.

## Supplementary Material

Supplementary figures and tables.

## Data Availability

The datasets were downloaded from the GEO database (https://www.ncbi.nlm.nih.gov/geo/). The data that support the findings of this study are available from the corresponding author.
