# Supplementary material for: Neutrophil extracellular traps prime the ZBP1-cGAS sensor complex, triggering necroptosis and inflammatory injury in acute pancreatitis
Source: Int J Biol Sci. 2026 Feb 4;22(5):2398–417. doi: 10.7150/ijbs.122290 (PMC12965145; doi:10.7150/ijbs.122290)
Supplement: Supplementary file 1 — Supplementary figures and tables. [file ijbsv22p2398s1.pdf]

Additional Files

Figure S1. Dynamically track of development of SAP. (A) Differential gene analysis, GO and KEGG enrichment analysis between SAP patients and MAP controls of GSE194331. (B) The building of mice AP models. (C) HE and IHC staining (Ly6g and F4/80) of mouse pancreas during development of SAP. Scale bar=200μm. (D) Typical images of WB analyses of PADI4 and CitH3 of mice pancreas during development of SAP. (E) Spearman correlation analysis among the expression of PADI4/CitH3 and histology score of pancreatic damage. (F) Typical images of WB analyses of PADI4 and CitH3 of pancreas in mice Control, Control+GSK484, SAP and SAP+GSK484 groups. (G) Serum cfDNA level of mice Control, Control+GSK484, SAP and SAP+GSK484 groups. The data are presented as the means ± SD (n=6 mice per group). Intergroup comparisons were evaluated using independent samples t-tests. Statistical significance was defined as $P < 0.05$.

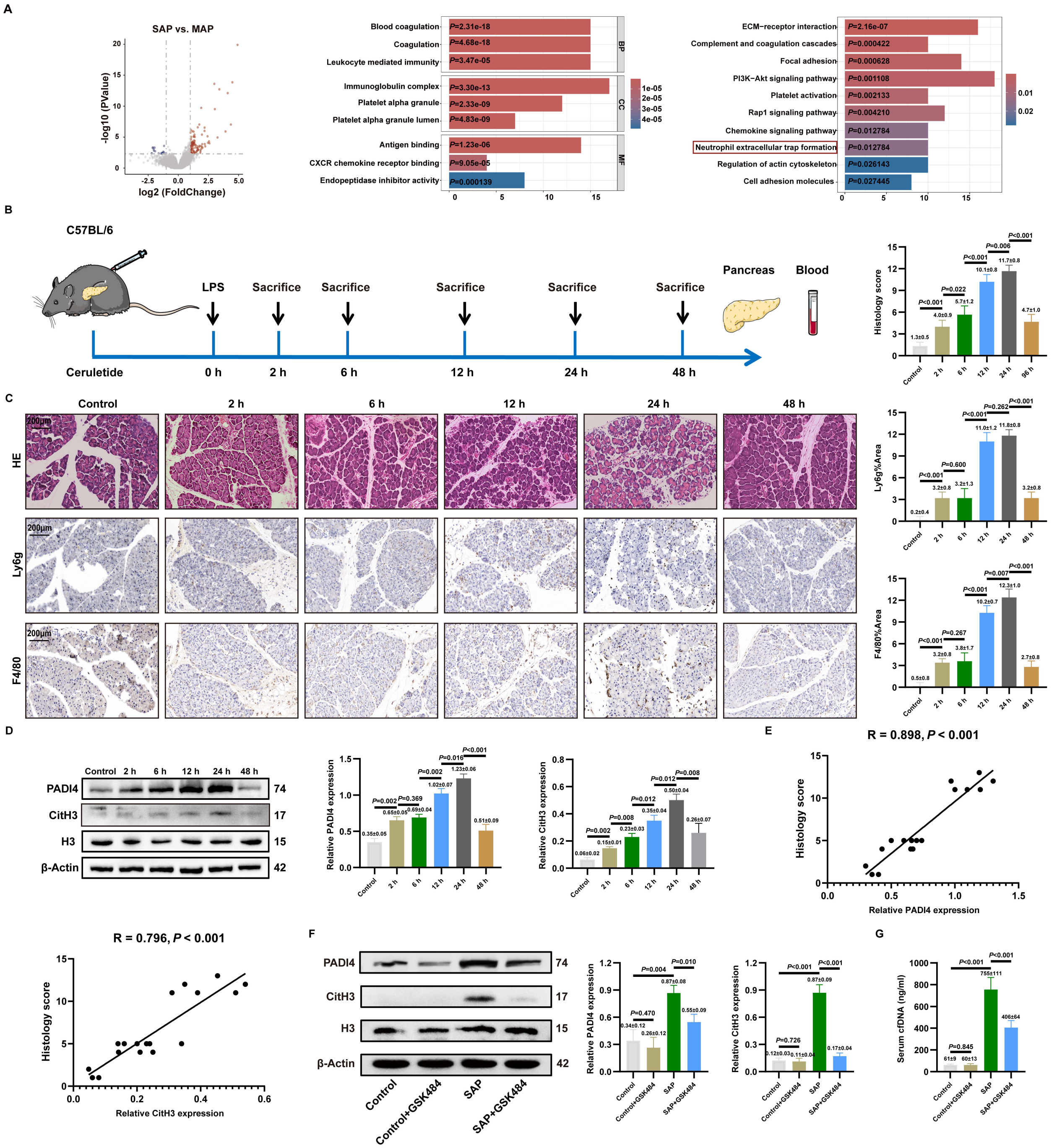

Figure S2. The concentration gradient of NETs for cellular SAP models and protective effect of DNase I in SAP models. (A) The NETs formation and the building of 266-6 AP models. (B) The level of cell death of 266-6 cells and cell viability of concentration gradient of NETs. (C) Expression levels of serum TNFα and IL6 of mice Control, Control+DNase I, SAP and SAP+ DNase I groups. (D) HE and IF (CitH3/MPO) of pancreas and serum cfDNA level in Control, Control+DNase I, SAP and SAP+DNase I groups, scale bar=100 μm and 50 μm. The data are presented as the means ± SD (n=6 mice per group, and n=3 cells per group). Intergroup comparisons were evaluated using independent samples t-tests. Statistical significance was defined as $P < 0.05$.

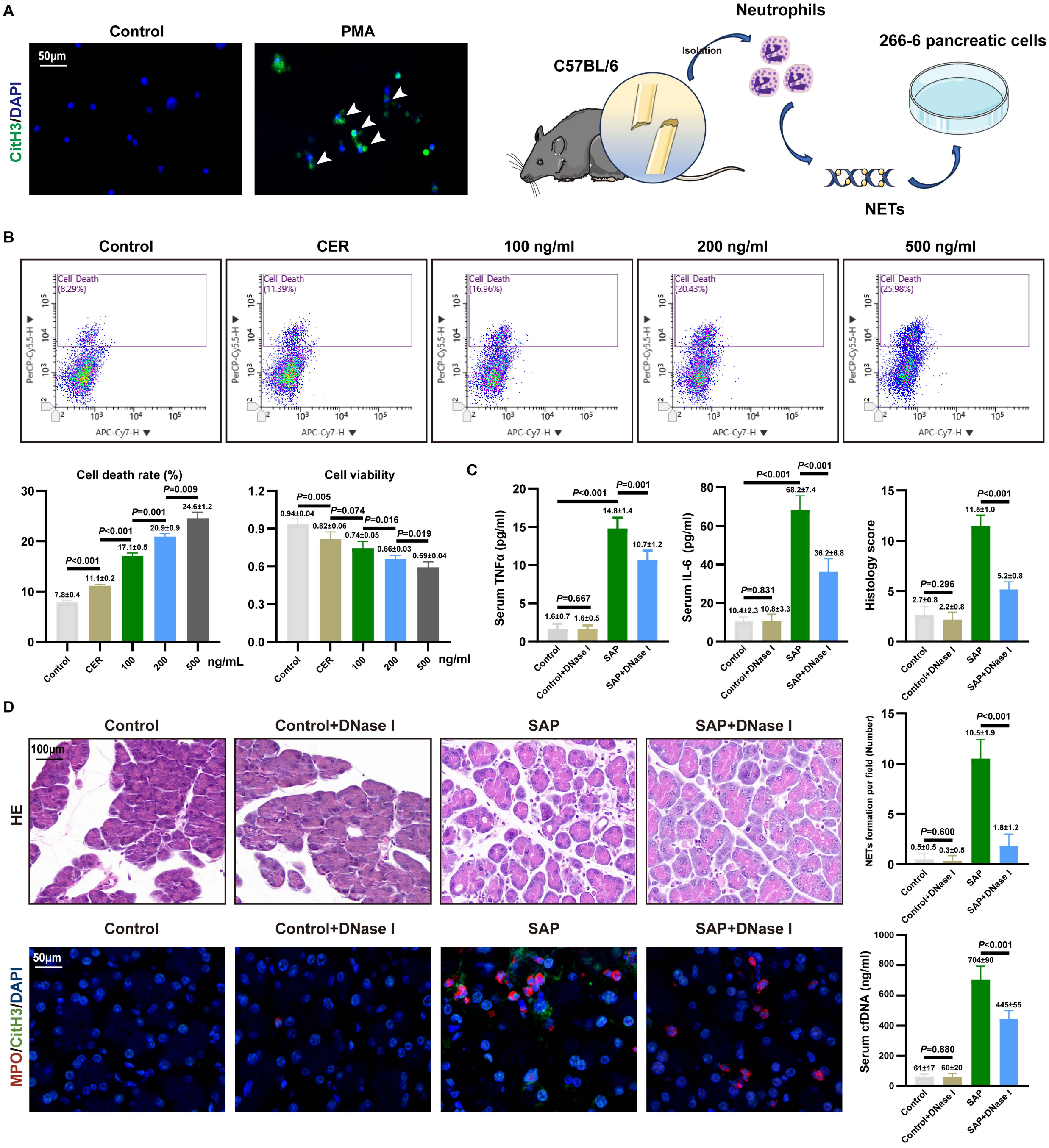

Figure S3. The activation of necroptosis and inflammatory pathway by NETs. (A) Heatmap and volcano map of mice pancreatic transcriptome sequencing data. (B) GSEA of NETs formation and multiple inflammatory pathways of mice pancreatic transcriptome sequencing data. (C) The level of cytosolic dsDNA in cellular Control, CER, CER+NETs, DNase I groups. (D) Typical images of WB analyses of cGAS, TBK1, p-TBK1, p65, p-p65, IRF3 and p-IRF3 in cellular Control, CER, CER+NETs, DNase I groups. (E) Typical images of WB analyses of RIPK3, p-RIPK3, MLKL and p-MLKL in cellular Control, CER, CER+NETs, DNase I groups. (F) The cell viability in cellular Control, CER, CER+NETs, DNase I and Dynasore groups. (G) Typical images of WB analyses of TBK1, p-TBK1, ZBP1, MLKL, p-MLKL and cGAS in cellular Control, NETs and Dynasore groups. The data are presented as the means±SD (n=3 cells per group). Intergroup comparisons were evaluated using independent samples t-tests. Statistical significance was defined as $P < 0.05$.

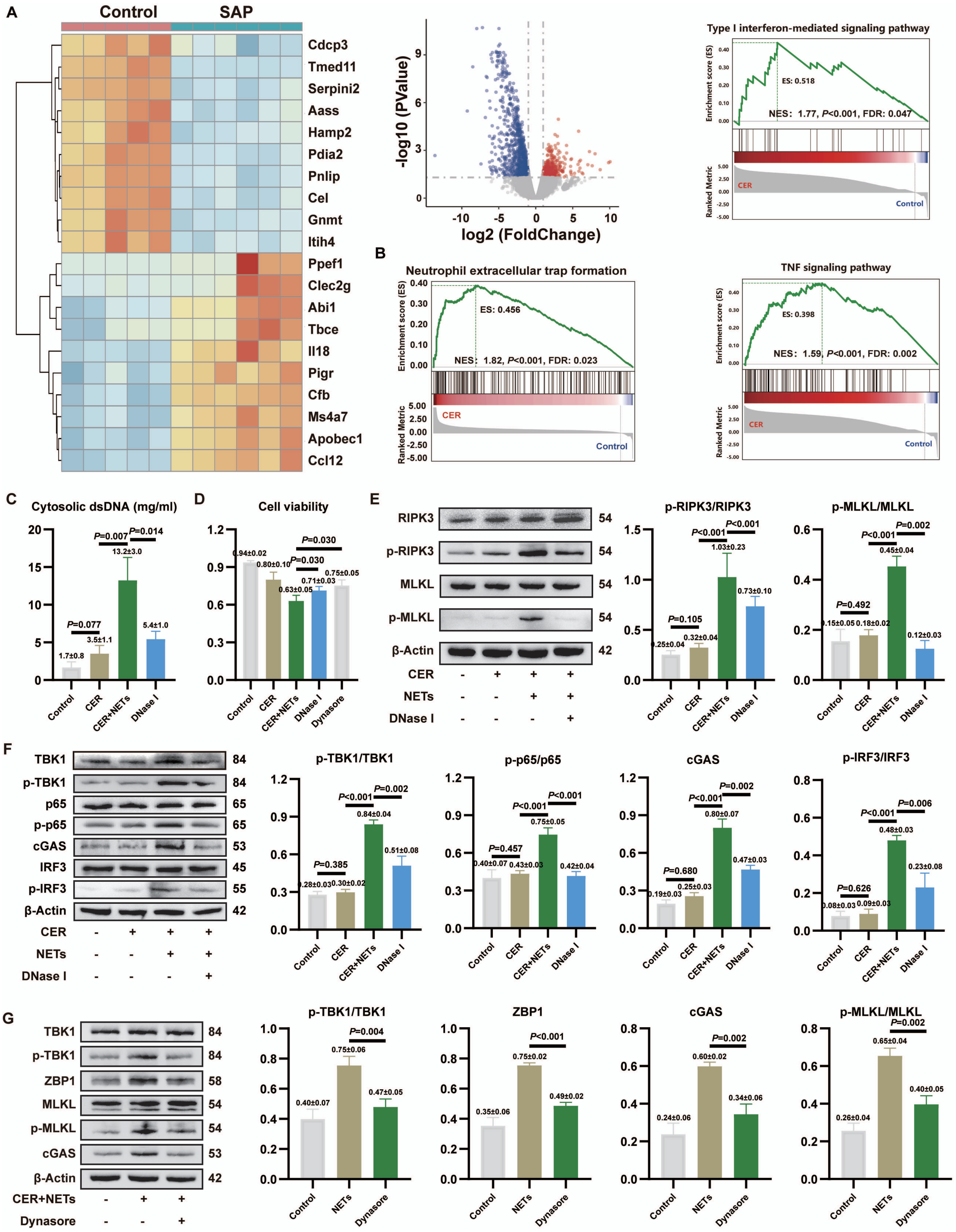

Figure S4. NETs are involved in the regulation of cGAS pathway activation and necroptosis. (A) The mRNA level of ZBP1 in mice transfected with AAV-sh-Zbp1 by RT-qPCR. (B) Typical images of WB analyses of ZBP1 in mice transfected with AAV-sh-Zbp1. (C) HE of pancreas in mice Control and SAP transfected with AAV-sh-Zbp1, scale bar=100 μm. (D) IF of GFP and MPO/CitH3 of pancreas in mice Control, Control+sh-Zbp1, SAP and SAP+ sh-Zbp1 groups. scale bar=50 μm. (E) Typical images of WB analyses of ZBP1, TBK1, p-TBK1, p65, p-p65, IRF3 and p-IRF3 in cellular Control, CER, NETs and Oe-Zbp1. (F) Typical images of WB analyses of cGAS, TBK1, p-TBK1, p65, p-p65, IRF3 and p-IRF3 in cellular Control, NETs, Oe-Zbp1 and Oe-Zbp1+Si- Cgas. The data are presented as the means±SD (n=6 mice per group, and n=3 cells per group). Intergroup comparisons were evaluated using independent samples t-tests. Statistical significance was defined as $P < 0.05$.

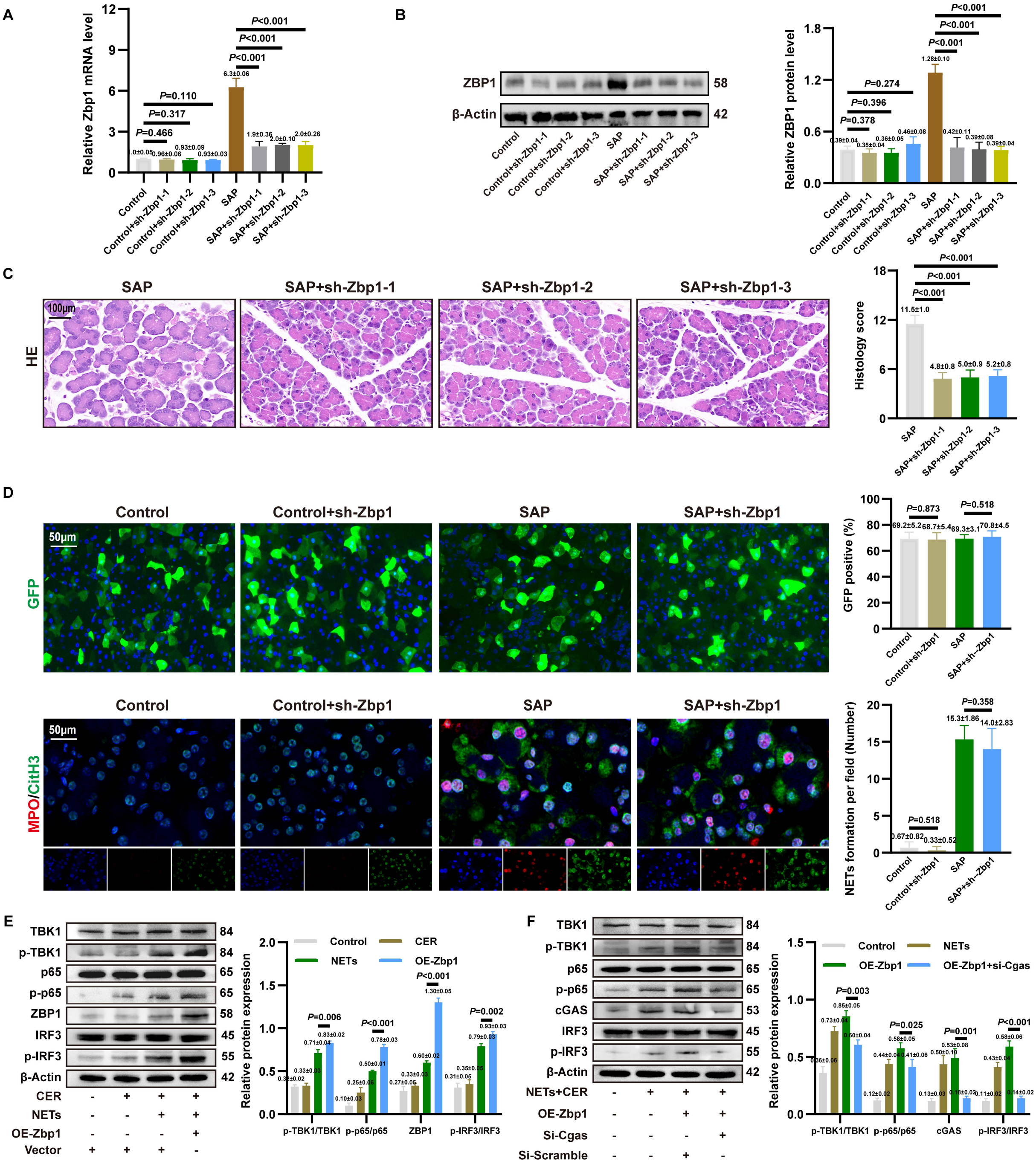

Figure S5. Apoptosis of mice pancreas of SAP. (A) HE and IHC staining (Caspase 8 and c-Caspase 3) of pancreas in mice Control, Control+GSK484, MAP, SAP and SAP+GSK484 groups. Scale bar=200μm (B) Typical images of WB of Caspase-8 and Caspase-3 of pancreas in mice Control, Control+GSK484, MAP, SAP and SAP+GSK484 groups. (C) HE and IHC staining (c-Caspase 3, Ly6g and F4/80) of pancreas in mice Control, SAP, SAP+Z-VAD and SAP+GSK872 groups, scale bar=100 μm. The data are presented as the means ± SD (n=6 mice per group). Intergroup comparisons were evaluated using independent samples t-tests. Statistical significance was defined as $P < 0.05$.

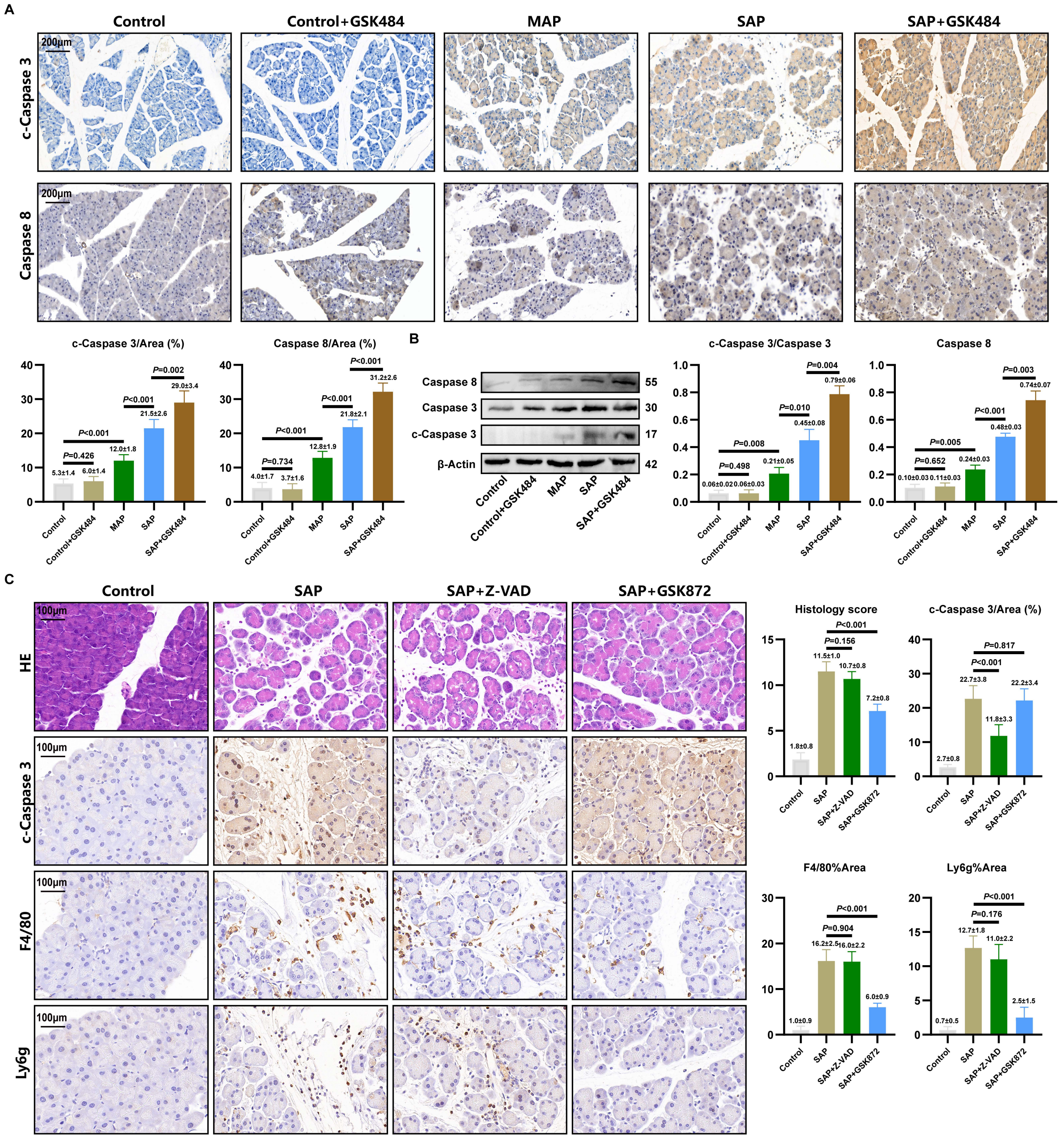

Figure S6. Molecular temporal dynamics of inflammatory and death signaling in vivo and in vitro. (A) IHC staining (TNFα and IFNβ) of mice pancreas at 2, 6, 12, 24 and 48 hours after the induction of AP model. (B) Typical images of WB analyses of p65, p-p65, ZBP1, MLKL, p-MLKL, cGAS, IRF3 and p-IRF3 of mice pancreas at 2, 6, 12, 24 and 48 hours after the induction of AP model. (C) Typical images of WB analyses of TBK1, p-TBK1, ZBP1, MLKL and p-MLKL at 6, 12 and 24 hours after the induction of cell AP models. The data are presented as the means ± SD (n=6 mice per group, and n=3 cells per group). Intergroup comparisons were evaluated using independent samples t-tests. Statistical significance was defined as $P < 0.05$.

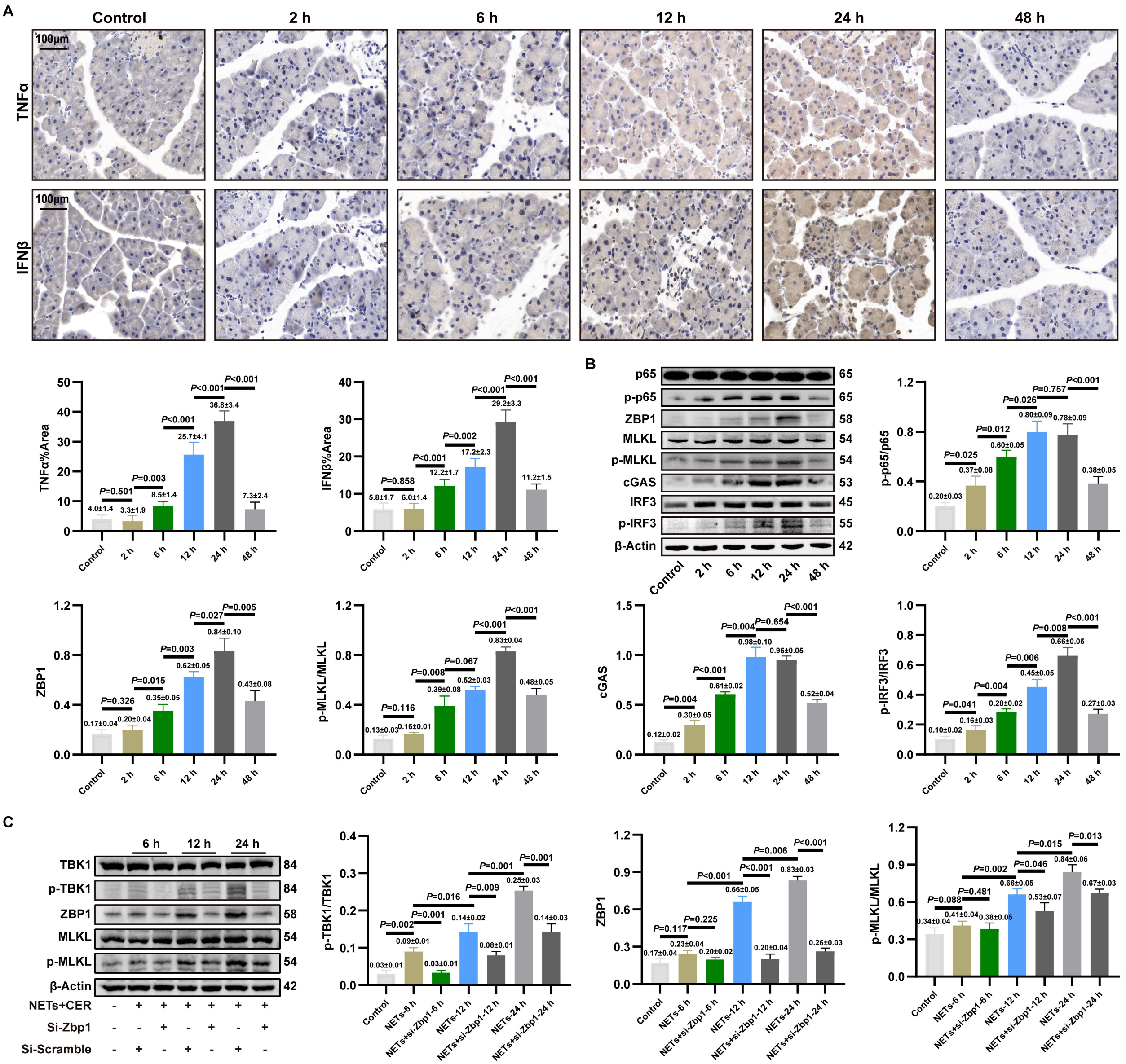

Figure S7. NETs induce mitochondrial damage. (A) IF image of CitH3 and dsDNA in cellular Control, CER, NETs, DNase I and Dynasore groups. Scale bar=10μm. (B) The copy numbers of cytosolic Mpo and Elane of cellular Control, NETs and DNase I gourps. (C) IF image of CitH3 and TOMM20 of pancreas in mice Control, SAP and SAP+GSK484 groups. Scale bar=50μm. (D) IF image of CitH3 and TOMM20 in cellular Control, NETs and DNase I groups. Scale bar=50μm. (E) The mPTP staining in cellular Control, NETs and DNase I groups. Scale bar=50μm. (F) Mitochondrial membrane potential levels in 266-6 cells by JC-1 staining in cellular Control, Control+Heparin, NETs and NETs+Heparin groups. Scale bar=50μm. (G) ROS levels of 266-6 cells by flow cytometry in Control, Control+Heparin, NETs and NETs+Heparin groups. The data are presented as the means ± SD (n=6 mice per group, and n=3 cells per group). Intergroup comparisons were evaluated using independent samples t-tests. Statistical significance was defined as $P < 0.05$.

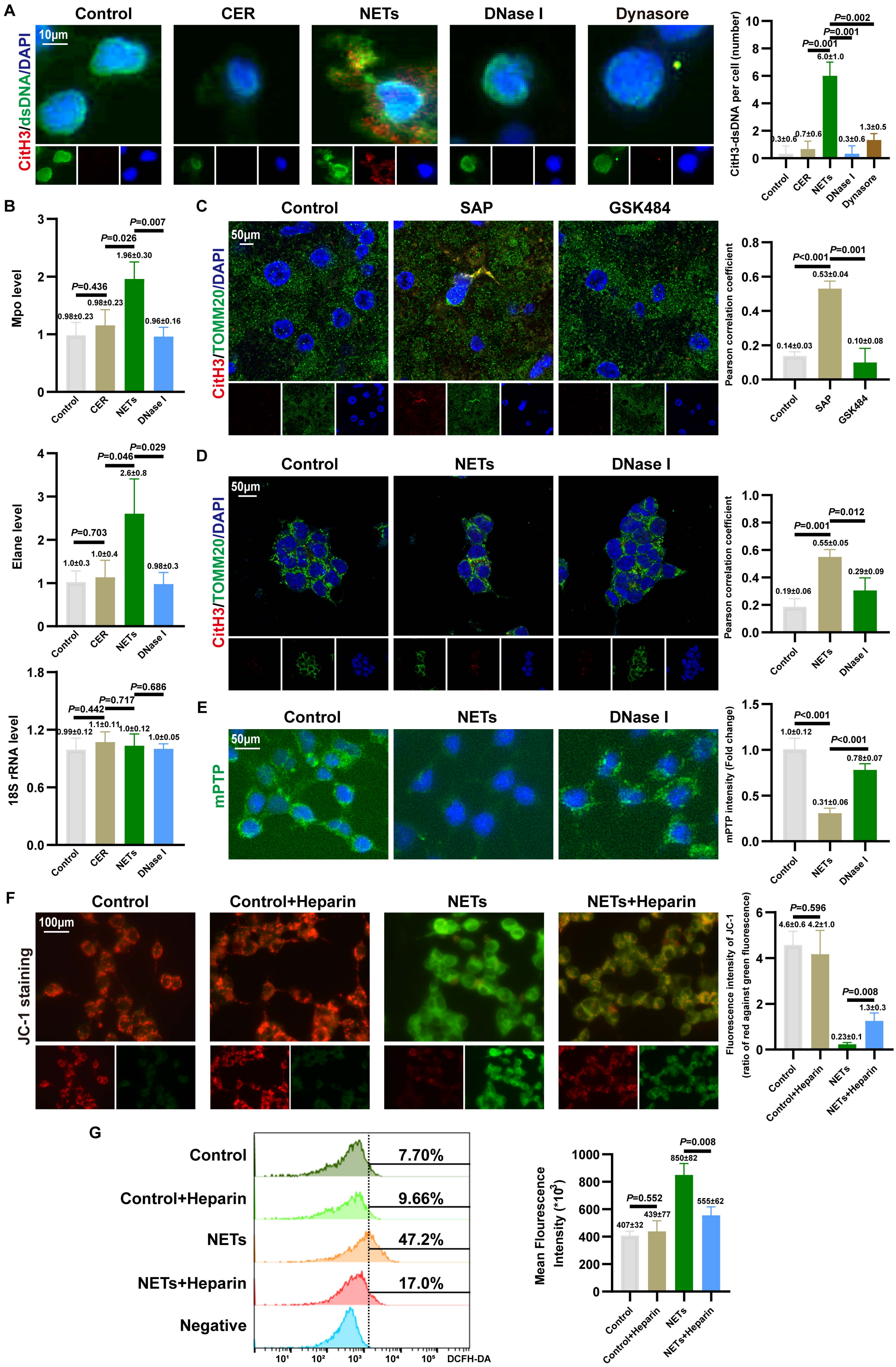

Figure S8. ZBP1-cGAS complex binds to mtDNA. (A) IF image of ZBP1/TFAM, cGAS/TFAM, Z-DNA/TFAM in cellular Control, NETs and DNase I. Scale bar=50μm. (B) IF image of GFP and MPO/CitH3 of pancreas in mice WT: Control, $Z\alpha^{mut}$: Control, WT: SAP and $Z\alpha^{mut}$: SAP groups. Scale bar=50μm. (C) IF image of Z-DNA/TFAM in mice pancreas and cellular AP models after Zα domain mutants. Scale bar=50μm. (D) IF image of Z-DNA/TFAM of pancreas in mice SAP and SAP+sh-Zbp1 groups. Scale bar=50μm. (E) IF image of Z-DNA/TFAM of pancreas in mice Control, Control+GSK484, SAP and SAP+GSK484 groups. Scale bar=50μm. The data are presented as the means ± SD (n=6 mice per group, and n=3 cells per group). Intergroup comparisons were evaluated using independent samples t-tests. Statistical significance was defined as $P < 0.05$.

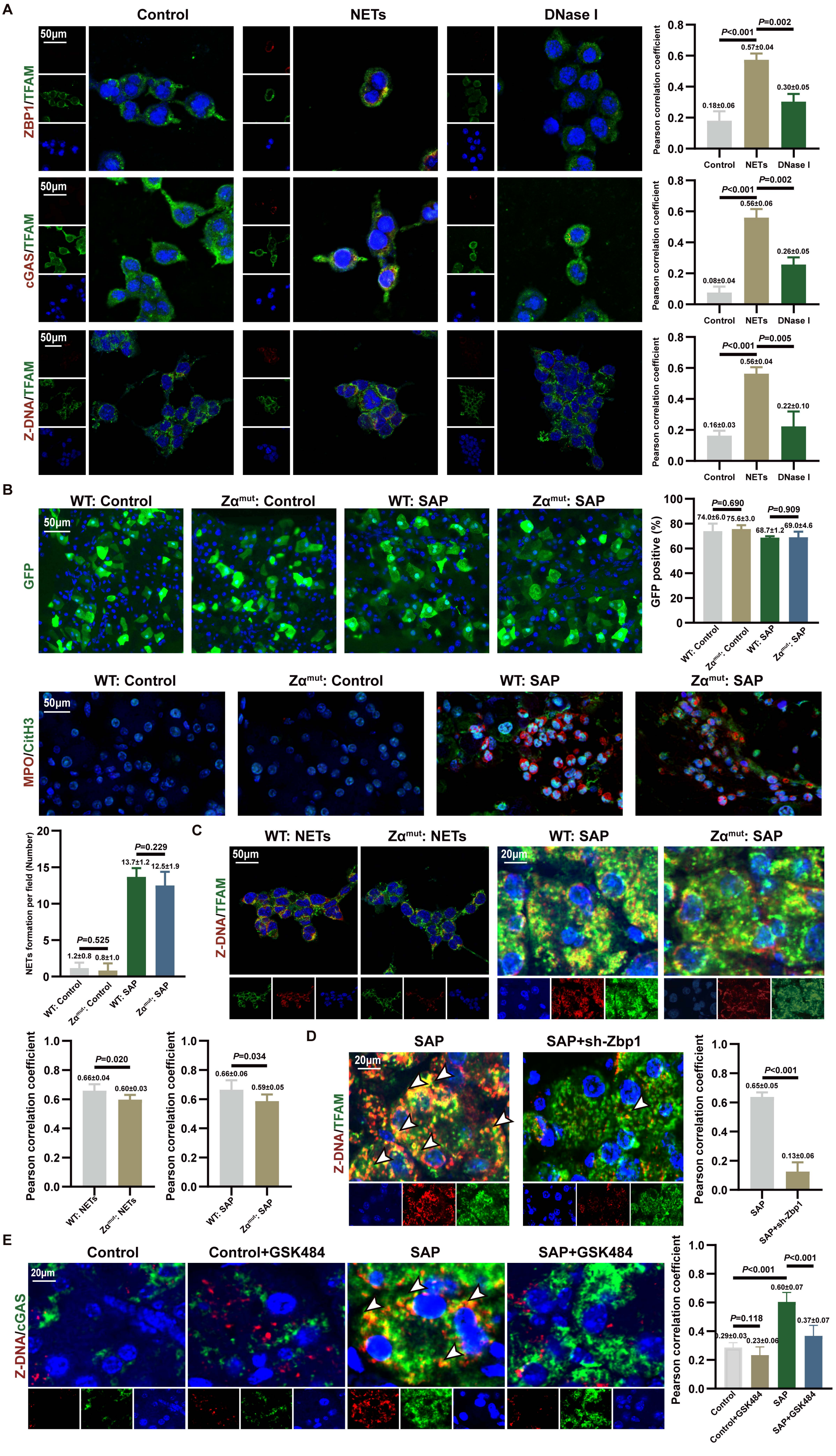

Figure S9. The NETs formation in SAP mice administrated with CsA. (A) IF image of CitH3 and MPO of pancreas in mice Control, Control+CsA, SAP, SAP+CsA groups. Scale bar=50μm. (B) Typical images of WB analyses of PADI4 and CitH3 of pancreas in mice Control, Control+CsA, SAP, SAP+CsA groups. The data are presented as the means ± SD (n=6 mice per group). Intergroup comparisons were evaluated using independent samples t-tests. Statistical significance was defined as $P < 0.05$.

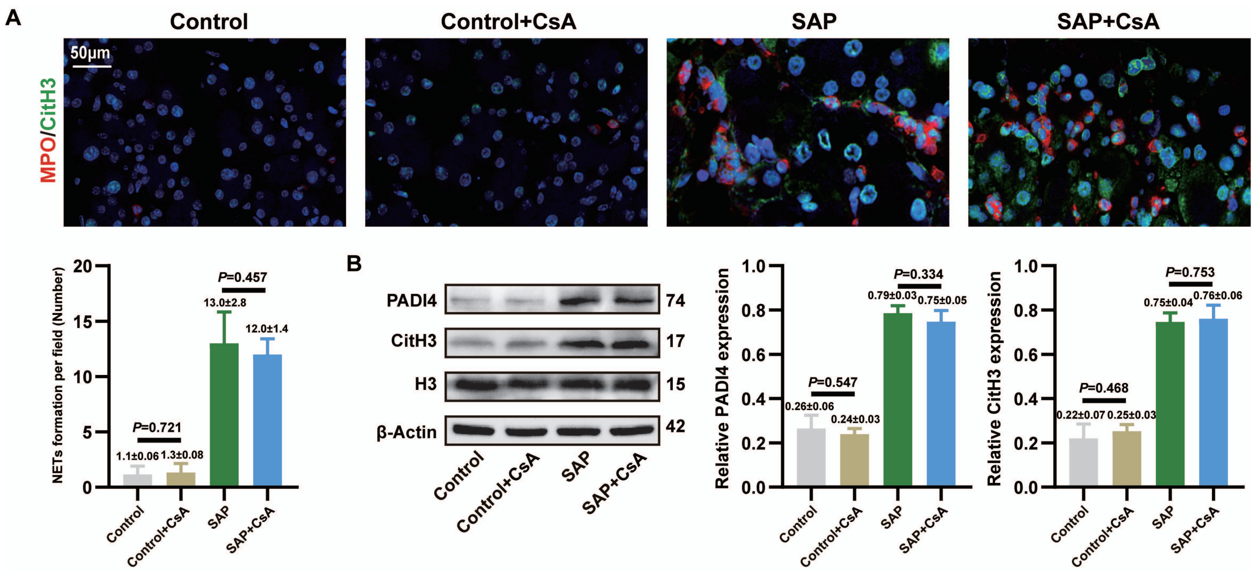

Table S1. Baseline characteristics of acute patients and healthy controls.

	Health (n=20)	MAP (n=20)	SAP (n=20)	*P* value
Sex, %				0.592†
Male	15 (75)	12 (60)	13 (65)	
Female	5 (25)	8 (40)	7 (35)	
Age, years	37.5±12.5	39.5±13.2	35.5±10.6	0.853*
BMI, kg/m^2	23.7±6.4	25.4±3.8	25.2±4.4	0.734*
Etiology, %				0.979†
Biliary		11 (55)	10 (50)	
Hyperlipidemia		5 (25)	5 (25)	
Alcoholic		3 (15)	4 (20)	
Others		1 (5)	1 (5)	
Coexisting condition, %				
Cardiovascular disease		5 (25)	6 (30)	
Chronic obstructive pulmonary disease		0	0	
Chronic renal insufficiency		0	1 (5)	
Diabetes		4 (20)	6 (30)	
Serum indicators within 24 h of onset				
WBC, × 10^9/L	4.8±1.2	9.5±1.5	10.1±2.1	<0.001*
N, × 10^9/L	2.7±1.0	7.0±0.8	7.6±0.8	<0.001*
HCT, %	40.0±5.5	38.0±4.0	38.5±3.5	0.314*
PLT, × 10^9/L	251±62	223±69	214±66	0.193*

† Data are from the χ^2 test;

* Data are from one-way ANOVA test;

Table S2. Antibodis.

Antibody	Provide vendor	Catalog number	Lot	Dilution
RIPK3	ABclonal	A27121	3600004178	1: 2000 (WB)
				1: 500 (IF)
p-RIPK3	Abcam	AB195117	1010164-39	1: 1000 (WB)
MLKL	Proteintech	66675-1-Ig	10027049	1: 2000 (WB)
p-MLKL	Abcam	AB196436	1001237-58	1: 2000 (WB)
ZBP1	Proteintech	13285-1-AP	00183169	1: 2000 (WB)
				1: 300 (IF)
TBK1	CST	12105T	LOT: 4	1:1000 (WB)
p-TBK1	CST	5483T	LOT: 15	1:1000 (WB)
p65	Selleck	F0006	F000601	1:1000 (WB)
p-p65	Selleck	F0155	F015502	1:1000 (WB)
cGAS	Proteintech	26416-1-AP	00181627	1:5000 (WB)
				1: 500 (IF)
IRF3	Abcam	AB68481	1130277-4	1:1000 (WB)
p-IRF3	Abmart	TA2436S	10296764	1:2000 (WB)
OPA1	ABclonal	A9833	5500022366	1:5000 (WB)
MFN2	ABclonal	A19678	4000000157	1:2000 (WB)
MFF	CST	84580T	LOT: 1	1:1000 (WB)
DRP1	ABclonal	A21968	3522120721	1:4000 (WB)
TFAM	ABclonal	A3173	3600004839	1: 500 (IF)
TOMM20	Abcam	AB56783	1131502-2	1: 50 (IF)
CitH3	Abcam	AB5103	1116477-8	1:1000 (WB)
CitH3	Abcam	AB281584	1095222-74	1:2000 (IF)
PADI4	Abcam	AB214810	1022494-29	1:1000 (WB)
Caspase 3	ABclonal	A19664	4000000143	1:5000 (WB)
				1:300 (IHC)
Caspase 8	Proteintech	13423-1-AP	00132978	1:1000 (WB)
				1:300 (IHC)
MPO	Proteintech	22225-1-AP	00133042	1: 300 (IF)
TNFα	Proteintech	60291-1-Ig	10026111	1: 500 (IHC)
IFNβ	ABclonal	A23651	5500028179	1: 100 (IHC)
Z-DNA	Absolute	AB00783-23.0	T2502B05	1:200 (IF)
dsDNA	Abcam	AB27156	1123857-2	1:200 (IF)
F4/80	Servicebio	GB113373	C781210220	1:500 (IHC)
Ly6G	ABclonal	A22270	3600006842	1:3000 (IHC)

Note: The specificity of all antibodies used in this study has been rigorously validated by the respective manufacturers, with supporting data and references available on their official websites.

Table S3. Sequence of siRNA, shRNA and primer.

Gene	Sequence (5’-3’)
Si-Zbp1	Sense: CUGUAUUCCAUGAGAAAUA(dT)(dT)<br>Antisense: UAUUUCUCAUGGAAUACAG(dT)(dT)
Si-Cgas	Sense: GGAUUGAGCUACAAGAAUAUU(dT)(dT)<br>Antisense: AAUAUUCUUGUAGCUCAAUCC(dT)(dT)
Sh-Zbp1	1. CTGTATTCCATGAGAAATA<br>2. GGACAAGTCCTTGCTCCAA<br>3. GCAACAAGATGACCATCCA
Oe-Zbp1	NM_021394.2
ZBP1 RHIM^{mut}	I192A/ Q193A /I194A /G195A
ZBP1 Zα^{mut}	N46A/ Y50A/ N122D/ Y126A
Zbp1	F: 5'-AAGAGTCCCCTGCGATTATTTG-3'<br>R: 5'-TCTGGATGGCGTTTGAATTGG-3'
mt-Nd1	F: 5'-CTAGCAGAAACAAACCGGGC-3'<br>R: 5'-CCGGCTGCGTATTCTACGTT-3'
mt-Cox1	F: 5'-GCCCCAGATATAGCATTCCC-3<br>R: 5'-GTTCATCCTGTTCCTGCTCC-3'
mt-D-loop	F: 5'- AATCTACCATCCTCCGTGAAACC-3<br>R: 5'- TCAGTTTAGCTACCCCCAAGTTTAA-3
18s rRNA	F: 5'-TAGAGGGACAAGTGGCGTTC-3'<br>R: 5'-CGCTGAGCCAGTCAGTGT-3'
Elane	F: 5'-TTGCCAGGAATTTCGTCATGT-3'<br>R: 5'-GTTGGCGTTAATGGTAGCGGA-3'
Mpo	F: 5'-AGTTGTGCTGAGCTGTATGGA-3'<br>R: 5'-CGGCTGCTTGAAGTAAAACAGG-3'
